# Supplementary material for: Synergistic binding sites in a metal-organic framework for the optical sensing of nitrogen dioxide
Source: Nat Commun. 2023 May 2;14:2506. doi: 10.1038/s41467-023-38170-9 (PMC10154382; doi:10.1038/s41467-023-38170-9)
Supplement: Supplementary file 1 — Supplementary Information [file 41467_2023_38170_MOESM1_ESM.pdf]

Supplementary Information

Synergistic Binding Sites in a Metal-Organic Framework for the Optical Sensing of Nitrogen Dioxide

Isabel del Castillo-Velilla,[1,‡] Ahmad Sousaraei,[1,‡] Ignacio Romero-Muñiz,[1] Celia Castillo-Blas,[1] Alba S. J. Méndez,[2] Freddy E. Oropeza,[3] Víctor A. de la Peña O'Shea,[3] Juan Cabanillas-González,[4] Andreas Mavrandonakis,*[5] Ana E. Platero-Prats*[1,6,7]

[1] Departamento de Química Inorgánica, Facultad de Ciencias, Universidad Autónoma de Madrid, Campus de Cantoblanco, 28049 Madrid, Spain.

[2] Deutsches Elektronen-Synchrotron DESY, Notkestraße 85, 22607 Hamburg (Germany)

[3] Photoactivated Processes Unit, IMDEA Energy, Parque Tecnológico de Móstoles, Avenida Ramón de la Sagra 3, 28935 Móstoles, Madrid, Spain.

[4] Madrid Institute for Advanced Studies, IMDEA Nanociencia, c/ Faraday 9, Campus de Cantoblanco, 28049 Madrid, Spain.

[5] Electrochemical Processes Unit, IMDEA Energy, Parque Tecnológico de Móstoles, Avenida Ramón de la Sagra 3, 28935 Móstoles, Madrid, Spain.

[6] Condensed Matter Physics Center (IFIMAC), Universidad Autónoma de Madrid, 28049, Campus de Cantoblanco, 28049 Madrid, Spain.

[7] Institute for Advanced Research in Chemical Sciences (IAdChem). Universidad Autónoma de Madrid, 28049 Madrid, Spain.

‡ These authors contributed equally to this work.

KEYWORDS. metal–organic frameworks • optical sensing • nitrogen dioxide • computational modelling • pair distribution function

Table of Contents

Supplementary Methods 2

Supplementary Note 1. Synthesis of the materials 4

Supplementary Note 2. ^{1}H Nuclear Magnetic Resonance (^{1}H-NMR) spectra 5

Supplementary Note 3. X-ray powder diffraction.. 6

Supplementary Note 4. Scanning Electron Microscopy. Energy Dispersing X-ray Spectroscopy.. 8

Supplementary Note 5. Nitrogen adsorption-desorption analyses 9

Supplementary Note 6. Thermal gravimetric analysis 10

Supplementary Note 7. ATR-FTIR analysis..12

Supplementary Note 8. Pair Distribution Function..13

Supplementary Note 9. X-Ray Absorption Spectroscopy..................................15

Supplementary Note 10. X-ray photoelectron spectroscopy.............................17

Supplementary Note 11. Computational methodology 19

Supplementary Note 12. Computational results..20

Supplementary Note 13. Optical sensing...25

Supplementary References..30

Supplementary Methods

All reagents were used as received from commercial suppliers unless otherwise stated.

Powder X-ray diffraction (PXRD) patterns were measured with a Bruker D8 diffractometer with a copper source operated at 1600 W, with step size = 0.02° and exposure time = 0.5 s/step. Samples were placed on a borosilicate sample holder and then the sample surface was levelled with a clean microscope slide. All the samples were ground prior to analysis unless otherwise stated. Data were measured using a continuous 2θ scan from 3.0-45º θ. For all samples, PXRD patterns are presented from 0–30º θ for visual clarity.

Scanning electron microscopy (SEM) images were collected with a JEOL JSM 7600F microscope, with field emission gun and electron detector in lens. And for the **energy dispersive X-ray spectra** (EDS) a S-3000N microscope equipped with an ESED and an INCAx sight of Oxford Instruments was used. All samples were prepared for SEM and EDS by dispersing the material onto a double sided adhesive conductive carbon tape that was attached to a flat aluminum sample holder and they were sputtered with carbon or gold (12 nm).

Nuclear magnetic resonance (NMR) spectra were acquired on a Bruker AV-300 spectrometer, running at 300 MHz for ^{1}H. Chemical shifts (δ) are reported in ppm relative to residual solvent signal with a value of 2.50 ppm for DMSO-d_6. ^{1}H digested solution NMR (100 µL D_2O, 1 mg NaF, 50 µL HF and 500 µL DMSO-d_6) of as-synthesized sample MOF-808.

Textural analyses. Nitrogen adsorption and desorption isotherms were measured at 77 K using a Micromeritics ASAP 2020 system. The samples were outgassed at 100 ºC for 16 h before the measurements. The specific surface areas (BET) were calculated by application of the Brunauer-Emmett-Teller equation taking the area of the nitrogen molecule as 0.162 nm^2. The linear range of the BET equation was located between 0.05–0.35 P/P_0, however, for all other materials studied due to their microporous natures this linear range was much narrower and displaced to lower relative pressures: P/P_0= 0.04–0.07. The micropore volume and external surface area, *i.e.* the area not associated with the micropores, were calculated using a t-plot analysis. Taking the thickness of an adsorbed layer of nitrogen as 0.354 nm and assuming that the arrangement of nitrogen molecules in the film was hexagonal close packed. The mesopore volumes of the materials were calculated from the volume of gas adsorbed at a relative pressure of 0.6 on the desorption branch of the isotherms, equivalent to the filling of all pores below 50 nm, minus the microporosity calculated from the corresponding t-plot. The total pore volume was calculated from the volume of gas adsorbed at a relative pressure of 0.95 on the adsorption branch of the isotherms. The pore-size-distribution (PSD) curves were obtained from the adsorption branches using non-local density functional theory (NLDFT) method for a cylinder pore in pillared clays, using a regularization of 0.100. MicroActive software was used to perform these analyses.

Thermogravimetric analyses and **differential thermal analyses** (TGA-DTA) were performed using a SDT Q600 from TA Instruments equipment in a temperature range between 20 °C and 800 °C in air (100 mL/min flow) atmosphere and heating rate of 10 ºC/min.

Infrared spectra (FTIR) were recorded on a PerkinElmer 100 spectrophotometer using a PIKE Technologies MIRacle Single Reflection Horizontal ATR Accessory from 4000-450 cm^{-1}.

Elemental analyses were performed with a LECO CHNS-932 analyser, with dry samples.

ICP analyses were performed with an Inducted Coupled Plasma Emission Spectrometer ICP PERKIN ELMER mod. OPTIMA 2100 DV. Samples (3 mg) were digested in 4 mL of a 1:1 H_2O_2:H_2SO_4 mixture (v:v) and taken to a 10 mL in a volumetric flask volume with distilled water.

Supplementary Note 1. Synthesis of the materials

MOF-808. Trimesic acid (210 mg, 1.0 mmol) and $ZrOCl_2\cdot8H_2O$ (970 mg, 3.0 mmol) were added to a mixture of 90 ml of formic acid (45 mL) and DMF (45 mL) in a screw cup glass bottle. The reaction was heated at 130 °C for 48 h in the oven. After cooling to room temperature, white powder was collected by centrifugation (12000 rpm, 2 min), and the solid was washed with DMF, distilled water and acetone (50 mL x 3 each) The solid was dried in the oven at 60ºC overnight. Then, the product (800 mg) was put in a solution 1 M of HCl (100 ml) stirring at room temperature for 24h, with the aim of exchanging some of the formates ligands for OH_2. The resulting mixture was centrifuged and the solid was washed with distilled water and acetone (50 mL x 3). The solid was dried in the oven at 60 ºC overnight yielding MOF-808 as a white powder (750 mg). **Elemental Analysis:** $[Zr_6O_8H_4(C_9H_3O_6)_2(COOH)_2(OH)_4(H_2O)_4]\cdot(C_3H_7NO)_{0.5}(H_2O)_{28}$ = $Zr_6C_{23}H_{73}N_1O_{54}$: Calcd.: C 13.85%, H 4.51 %, N 0.38%; *Found*: C 13.23%, H 4.21%, N 1.07%. **^{1}H-NMR:** 8.63 (s, 6H, 2 × BTCs), 8.11 (s, 1.9H, 1.7 × HCOO-), 2.87 (s, 1H, 0.3 × DMF), 2.72 (s, 1H, 0.3 × DMF).

Cu-MOF-808. Different salts of Cu (II) and Cu (I) were tested as precursors to introduce the copper in the MOF structure. They were characterized by Powder X-ray diffraction (Fig S4.1) and ICP (Table S2.1) obtaining as the best option the copper (II) acetate. The explanation relies on the basicity of the conjugate pair of the salts, as acetate is the most basic pair it is the best one to equilibrate the positive charges of the structure formed in the removal of formates at the activation.

Supplementary Table 1. Evaluation of different copper precursors for the synthesis of Cu-MOF-808.

Copper salt	*Ratio MOF:Cu salt*	*Atoms of Cu per cluster*	*Yield of incorporation*	*Observations*
$Cu(CH_3COO)_2$	1:5	3.25	65 %	Turquoise powder
$Cu(NO_3)_2$	1:5	0.13	2.7 %	White powder
$CuCl_2$	1:5	0.00	0.0 %	White powder
CuI	1:5	0.02	0.4 %	Brown powder

Thus, we chose copper (II) acetate as precursor for the metalation, following the procedure below. A mixture of MOF-808 treated with HCl (100 mg, 0.056 mmol) and $Cu(CH_3COO)_2\cdot H_2O$ (68 mg, 0.34 mmol) and MeOH (10 mL) was placed in a sealed vial. The reaction was stirred at 60 °C overnight. After cooling to room temperature, the reaction mixture was centrifuged, and the solid was washed with MeOH, distilled water and acetone (3 times with 10 mL each). The solid was dried in the oven at 60 ºC overnight yielding Cu-MOF-808 as a turquoise powder (85 mg). **ICP analyses**, ratio Cu/Zr_6: 3.3. $[Zr_6Cu_{3.3}O_8H_4(C_9H_3O_6)_2(OH)_{9.1}(H_2O)_{3.2}(C_2H_3O_2)_{3.5}]\cdot(C_3H_7ON)_{0.4}$ = $Zr_6Cu_{3.3}C_{26.2}H_{38.8}N_{0.4}O_{39.7}$ **Elemental analyses:** *Calcd.*: C 17.97%, H 2.23%, N 0.32%; *Found:* C 19.50%, H 2.61%, N 0.20%. **^{1}H-NMR:** 8.62 (s, 6H, 2 × BTCs), 2.85 (s, 0.5H, 0.17 × DMF), 2.73 (s, 0.5H, 0.17 × DMF), 1.89 (s, 9H, 3 x acetates).

Water molecules are removed from the pores drying in the oven. However, over time these molecules can be re-adsorbed in the pores.

Supplementary Note 2. [1]H Nuclear Magnetic Resonance (^{1}H-NMR) spectra

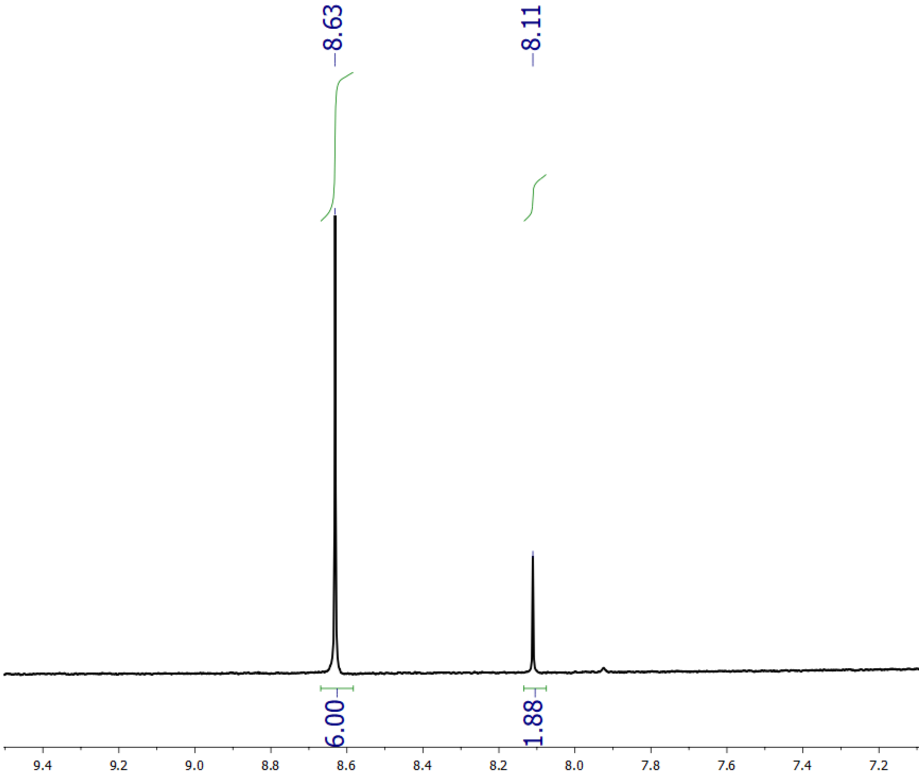

Supplementary Figure 1. ^{1}H NMR spectrum of MOF-808 after treatment with HCl in DMSO-d_6, 300 MHz.

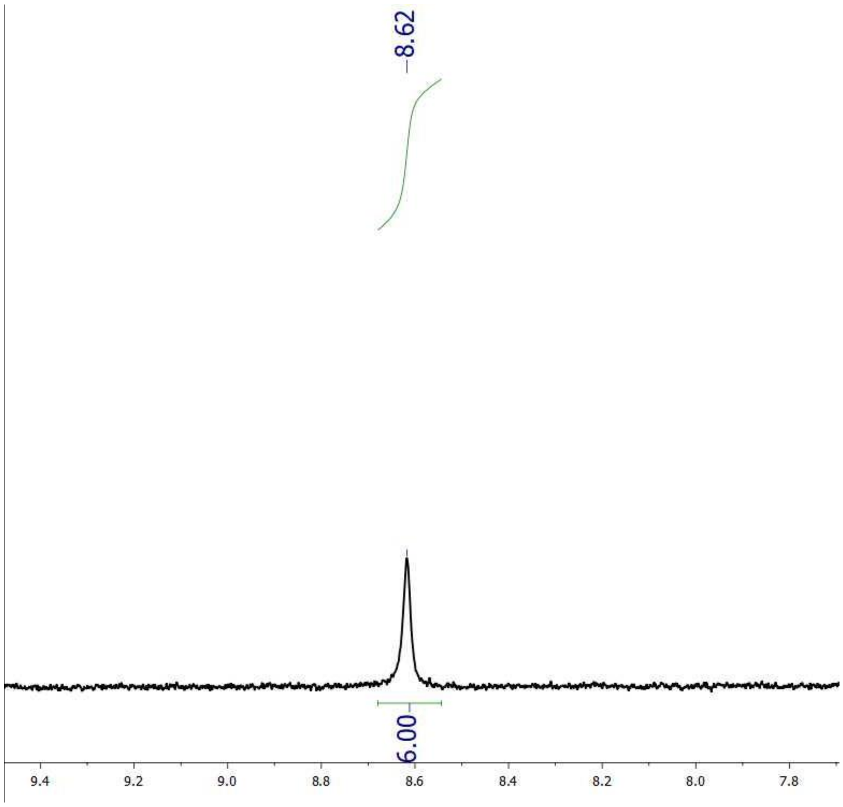

Supplementary Figure 2. ^{1}H NMR spectrum of Cu-MOF-808 in DMSO-d_6, 300 MHz.

Supplementary Note 3. X-ray powder diffraction

X-ray diffraction patterns of the materials show a high crystallinity and pure phases compared with the calculated pattern from single crystal data.[1]

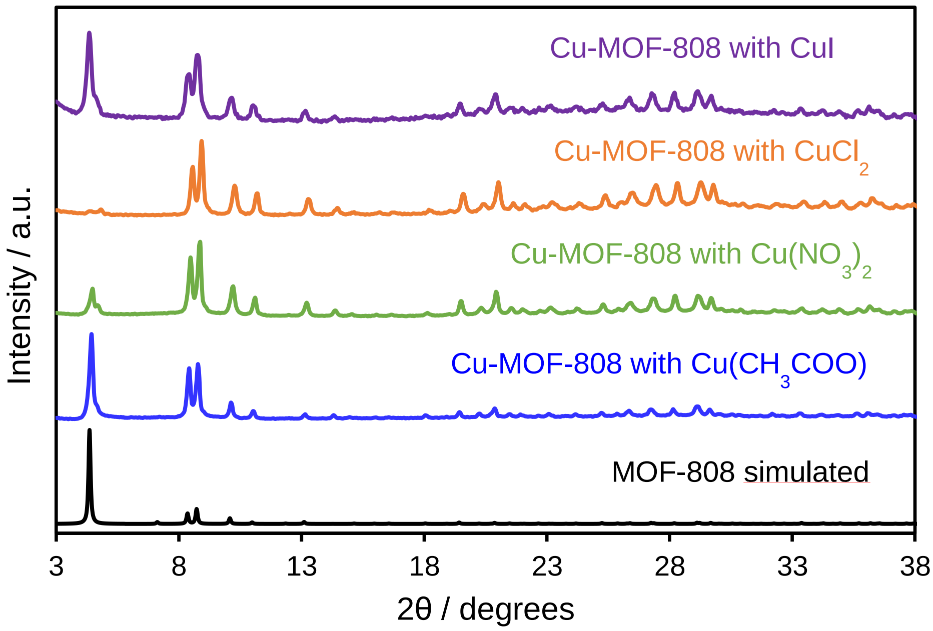

Supplementary Figure 3. X-ray powder diffraction patterns of possible Cu-MOF-808 materials and their comparison with the calculated MOF-808 reported by Yaghi et al.[1]

Results of Pawley refinements are shown in Table S4.1 and Fig S4.3.

Supplementary Table 2: Pawley refinement values for synthetised samples.

Sample	*a*	*Rwp*	*Rp*	*Zero*	*U*	*V*	*W*
MOF-808	35.04771	10.43	7.64	3.73	28836.98	-8954.14	423.385
Cu-MOF-808	35.01827	8.37	5.28	3.90	8745.79	-2553.80	225.79

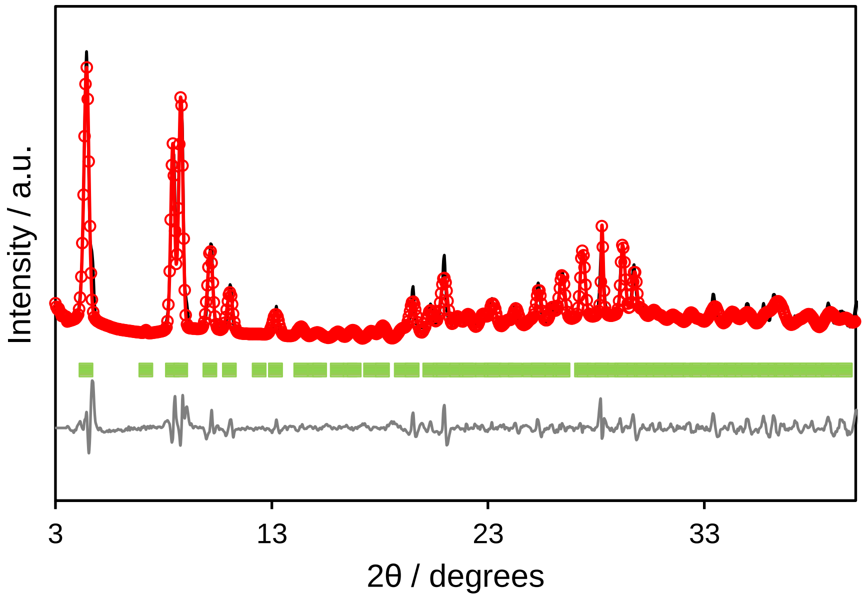

Supplementary Figure 4. Pawley refinement for MOF-808 activated.

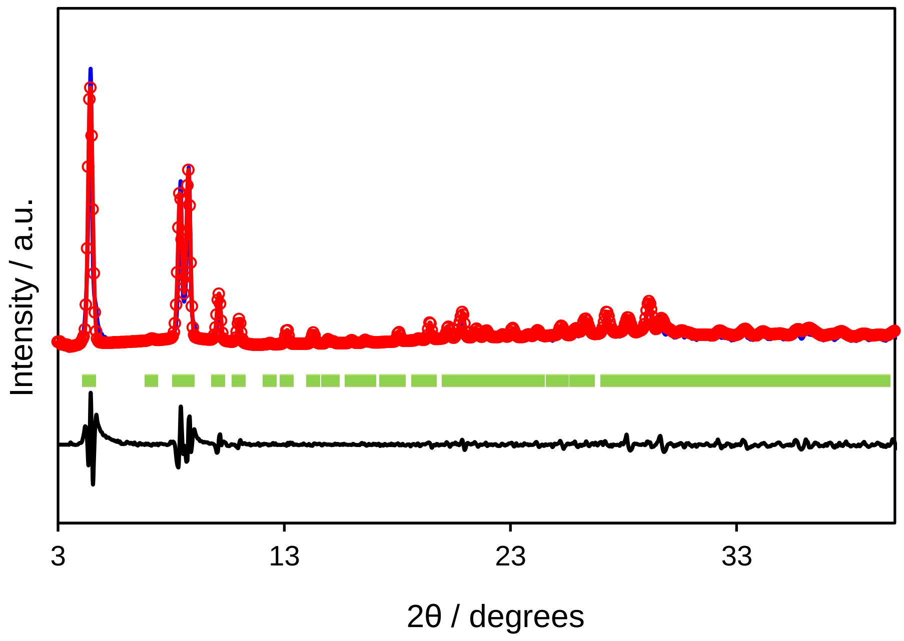

Supplementary Figure 5. Pawley refinement for Cu-MOF-808.

Supplementary Note 4. Scanning Electron Microscopy. Energy Dispersing X-ray Spectroscopy

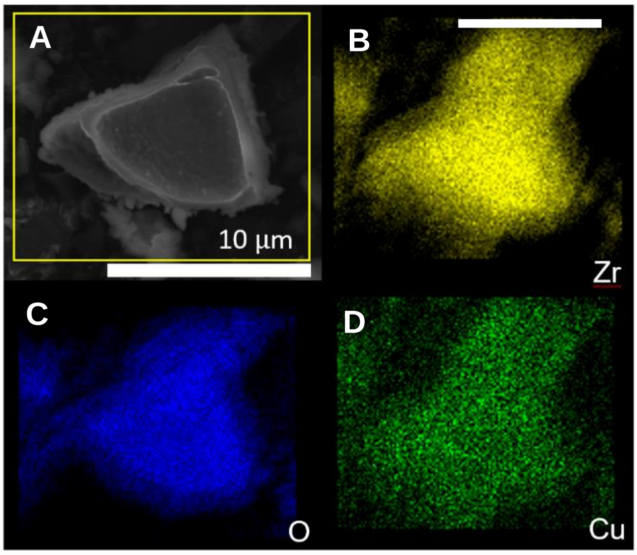

Supplementary Figure 6. (A) Image of a crystal of Cu-MOF-808 and corresponding Energy Dispersing X-ray (EDX) spectroscopy mappings of (B) Zr, (C) O and (D) Cu.

Mapping images show the presence of all the elements in the crystallites. Zirconium and Copper are homogeneously distributed in the octahedral crystals.

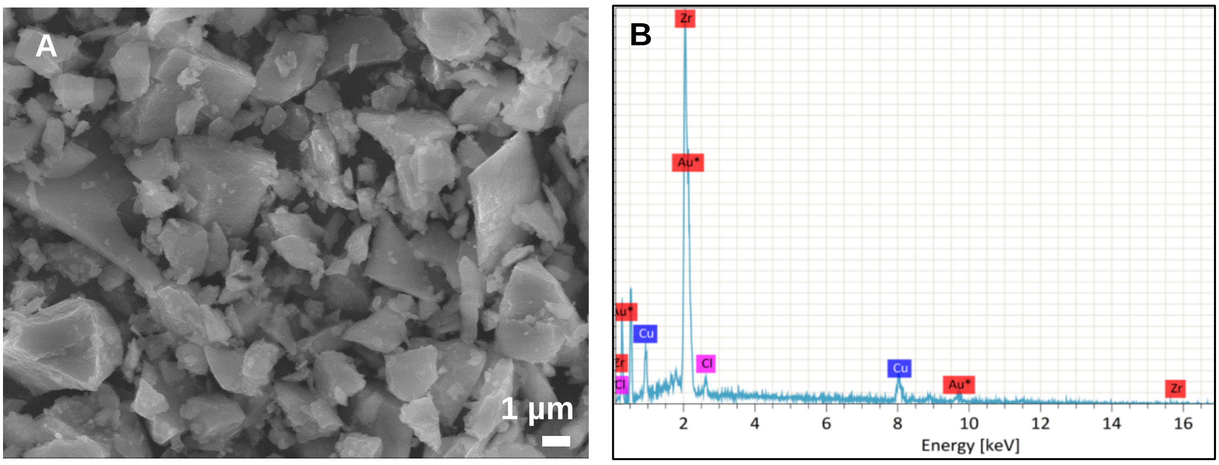

Supplementary Figure 7. (A) SEM image of a Cu-MOF-808 sample and (B) corresponding EDS spectrum.

SEM images show crystallites with a size between 3-5 µm. Mapping images show the presence of all the elements in the crystallites, Zirconium and Copper are homogeneously distributed.

Supplementary Note 5. Nitrogen adsorption-desorption analyses

The BET analysis of the materials show a diminution of the specific surface area of metalated materials. Regarding the NLDFT pore size distribution calculation, the Cu-loaded MOF seems to maintain the micropore window (*ca.* 12 Å and *ca.*18 Å) but the micropore contribution (taken as the pore width under 24 Å) to the total pore volume rapidly decreases with the metal loading.

Supplementary Table 3: Data collected from N_2 isotherms at 77 K, BET and t-plot analysis.

Sample	Heat treatment	Surface area (BET) / m^2g^{-1}	External area / m^2g^{-1}	Micropore volume / cm^3g^{-1}	Mesopore volume / cm^3g^{-1}	Total pore volume / cm^3g^{-1}
MOF-808	100ºC	1063.27	61.85	0.35	0.01	0.52
Cu-MOF-808	100ºC	520.41	61.79	0.18	0.02	0.30

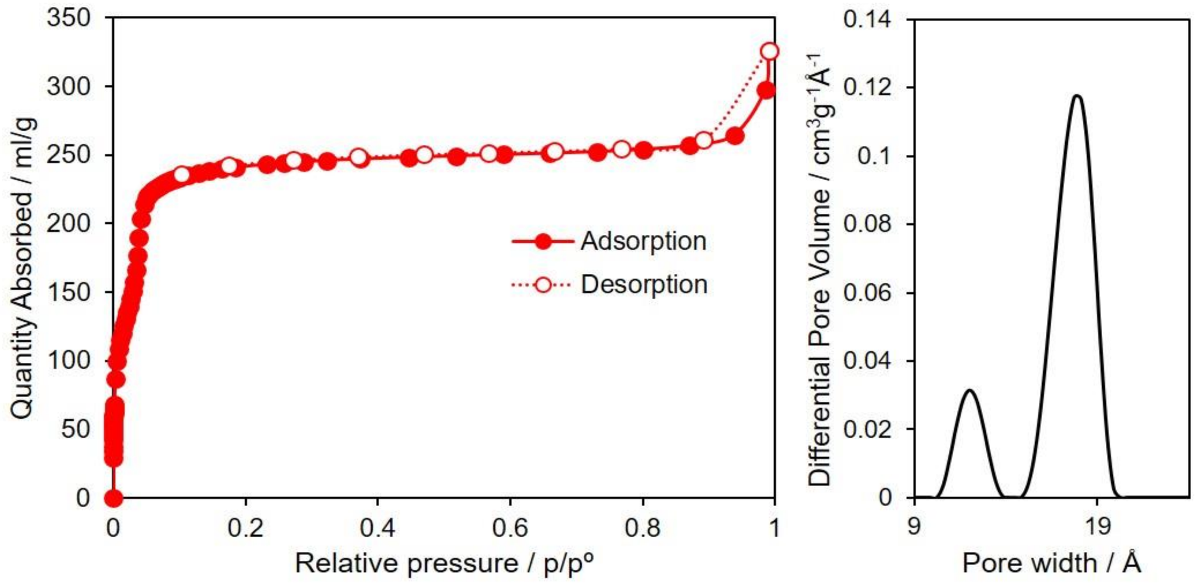

Supplementary Figure 8. N_2 isotherm and DFT pore size distribution of MOF-808 pristine.

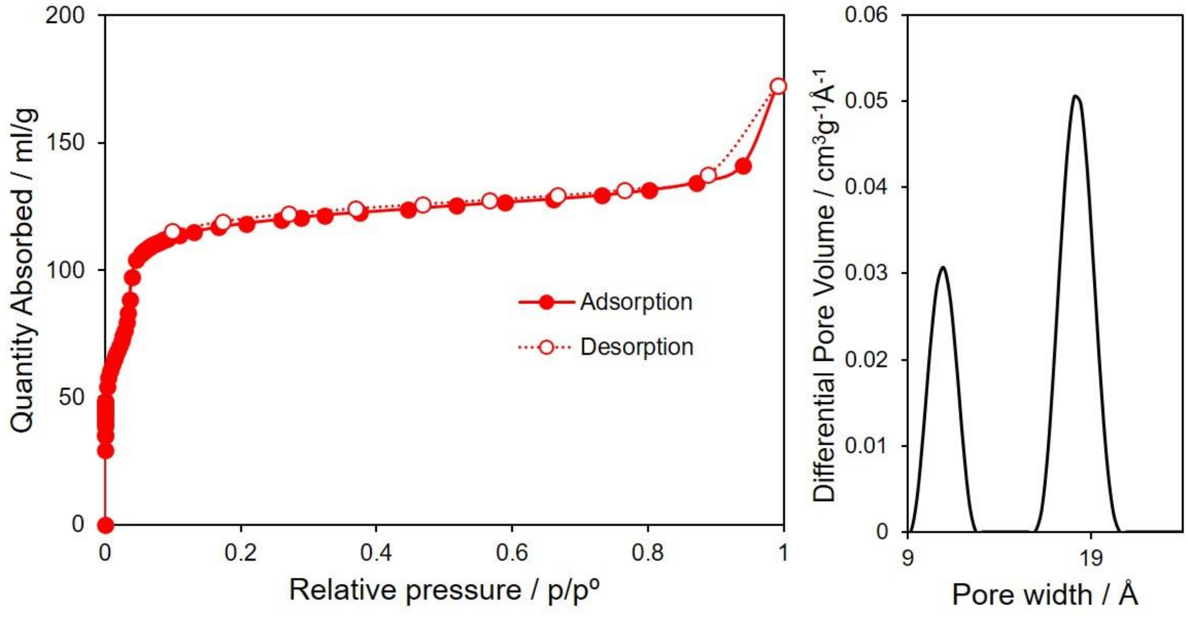

Supplementary Figure 9. N_2 isotherm and NLDFT pore size distribution of Cu-MOF-808.

Supplementary Note 6. Thermal gravimetric analysis

TGA data collected on the materials showed first a mass loss associated with loss of water molecules with values of 32.5% and 31.1% for MOF-808 and Cu-MOF-808, respectively. This first curve is more pronounced in MOF-808, than Cu-MOF-808 due to the amount of water which this material possesses (Fig. S7.1, Fig. S7.2 and Fig. S7.3).

For MOF-808 in the temperature range of 200-600 ºC, there is a 30.0 % weight loss corresponding to the decomposition of BTC ligands, formates and the trapped DMF (calculated 32.2 %). And all weight loss from 20 ºC to 800 ºC is 62.6 % which matches the destruction of MOF-808 to the residual of this material, ZrO_2 (calculated 62.3 %) (Fig S7.2).

For Cu-MOF-808 in the temperature range of 200-600 ºC, there is a 25.7 % weight loss corresponding to the decomposition of BTC ligands (calculated 23.9 %). And the weight loss from 20 ºC to 800 ºC is 56.8 %, attributing the remaining ZrO_2 and CuO (calculated 55.1 %) (Fig. S7.3).

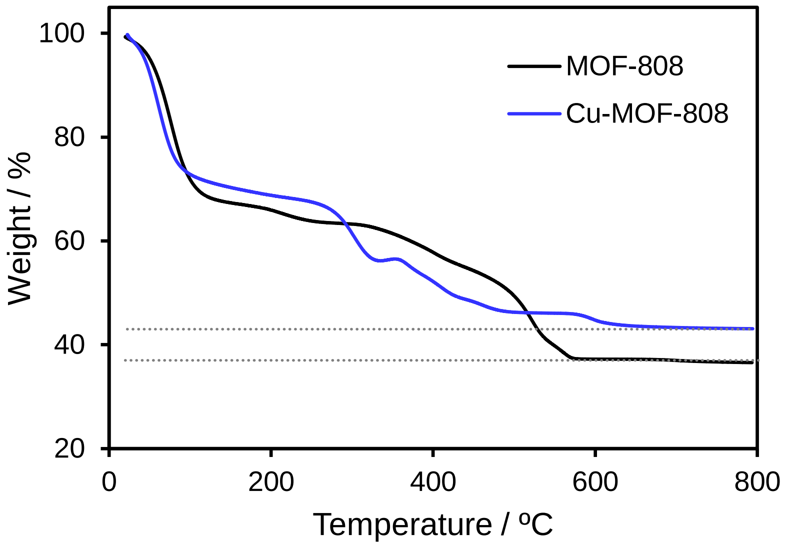

Supplementary Figure 10. Thermogravimetric analyses of materials.

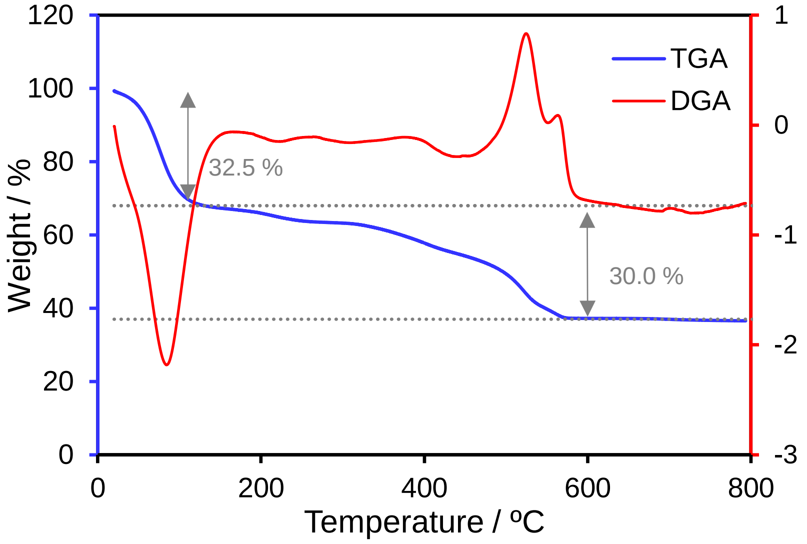

Supplementary Figure 11. Thermogravimetric analysis and differential thermal analysis of MOF-808.

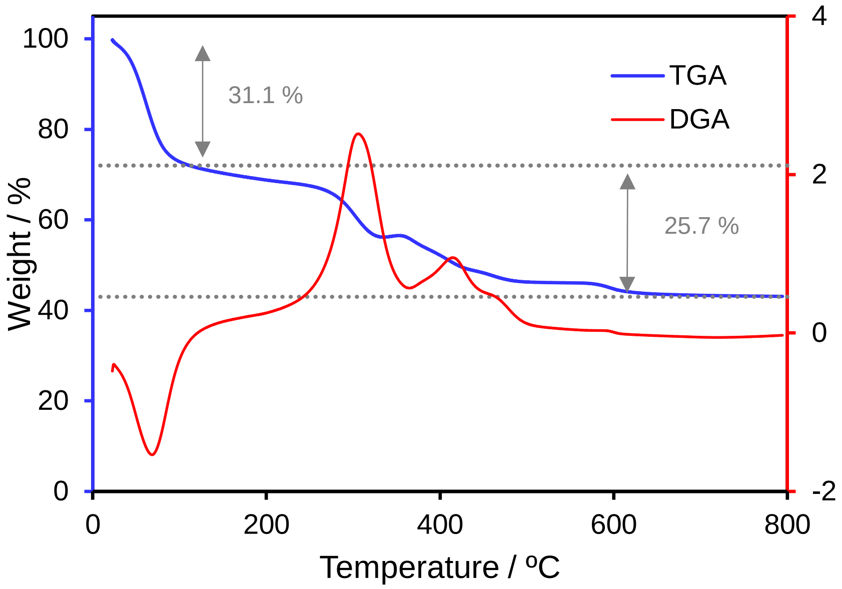

Supplementary Figure 12. Thermogravimetric analysis and differential thermal analysis of Cu-MOF-808.

Supplementary Note 7. ATR-FTIR analysis

The FTIR analysis shows the signals of principal functional groups of the structure. The Cu-MOF-808 FTIR spectra before and after NO_2 sensing process are compared. No significant difference is remarked between them. The vibrational band assignment was made based on previous work.[2]

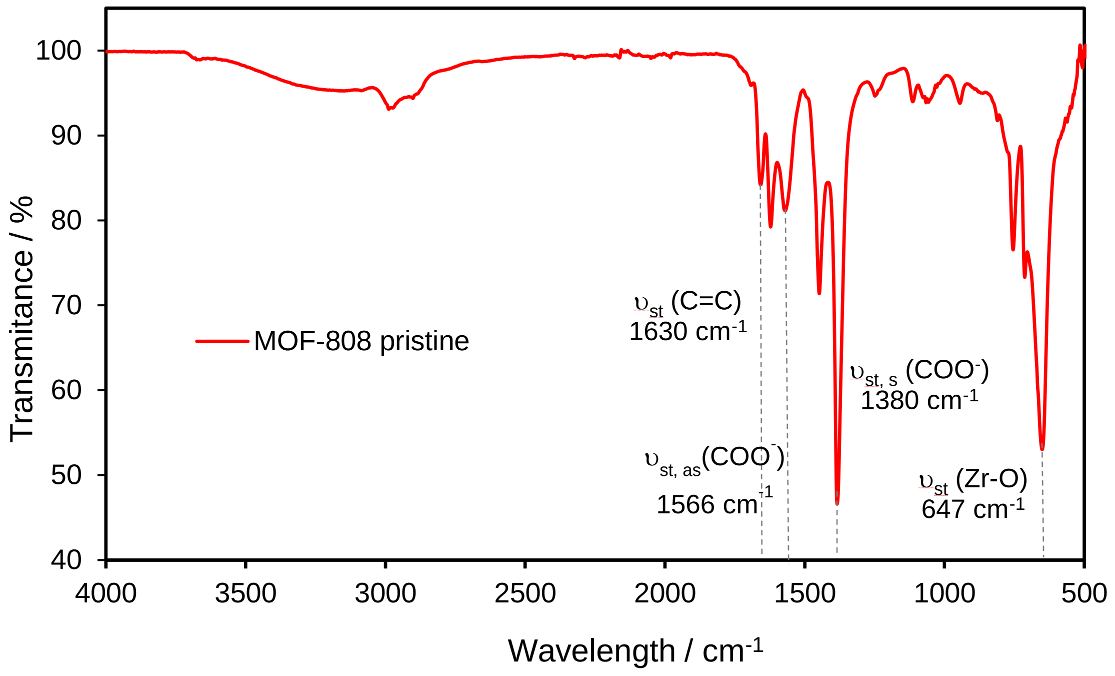

Supplementary Figure 13. ATR-FTIR of MOF-808 pristine after activation with HCl.

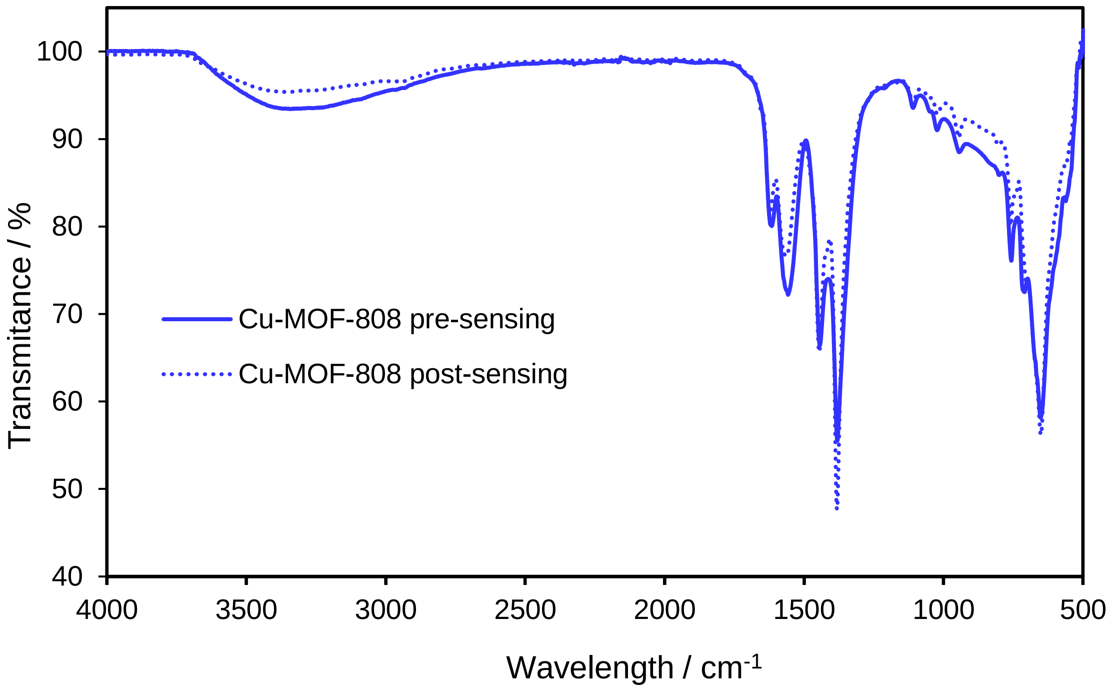

Supplementary Figure 14. ATR-FTIR of Cu-MOF-808 before (solid) and after NO_2 exposure (dashed).

Supplementary Note 8. Pair Distribution Function

Synchrotron X-ray total scattering data suitable for PDF analyses were collected at the P02.1 beamline at PETRA III (Deutsches Elektronen-Synchrotron) using 60 keV (0.207 Å) X-rays. Samples were loaded in polyamide (kapton) capillaries (0.8 mm Ø) and sealed using epoxy. Data were collected using an amorphous silicon–based Varex XRD 4343CT (150×150 μm^2 pixel size, 2880 x 2880 pixel area, CsI scintillator directly deposited on amorphous Si photodiodes) area detector. Geometric corrections and reduction to 1D data used DAWN Science software.[3] PDFs were obtained from the data within PDFgetX3 within xPDFsuite to a Qmax = 22 $Å^{-1}$.[4] Differential PDFs were obtained by subtraction of a reference PDF (pristine MOF-808) from Cu-MOF-808 in real space after applying a normalization factor to the data.

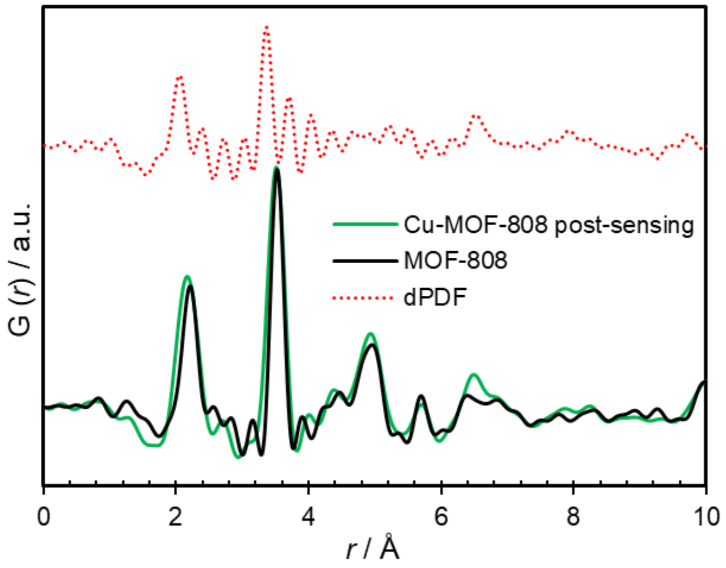

Supplementary Figure 15. PDF data for Cu-MOF-808 after sensing measurement. In red is the dPDF data for Cu-MOF-808.

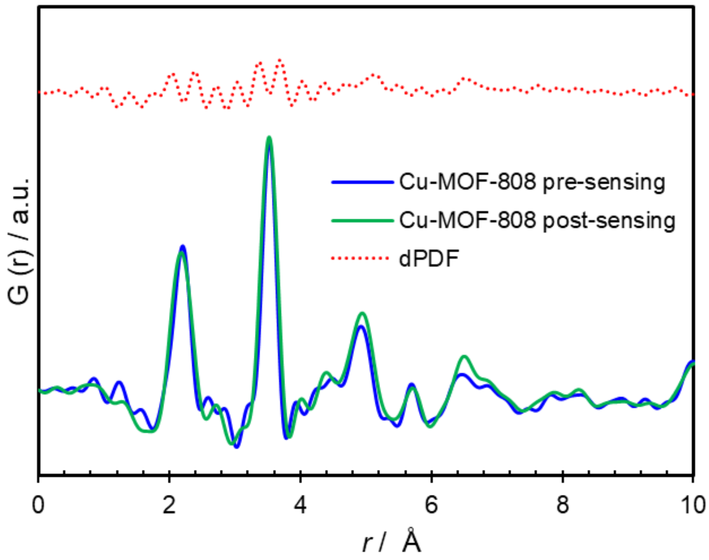

Supplementary Figure 16. PDF data for Cu-MOF-808 materials before and after sensing measurement. In red the differential PDF data.

The PDF characterization after sensing does not show any significant new distance in Cu-MOF-808. This indicates that the material is stable to the sensing process and also corroborates the theory that the interaction with NO_2 is reversible.

Supplementary Note 9. X-Ray Absorption Spectroscopy

Transmission and fluorescence geometry XAS measurements were performed at the P65 beamline of PETRA III. Cu *K*-edge XAS spectra were acquired from 8975 to 9010 eV, resulting in a k-range up to 12 Å^{-1}. The data analysis and background removal were performed within ATHENA and ARTEMIS.[5] $Cu(CH_3CO_2)_2$ and CuO were employed as references. The data were collected at 10 K using a He cryostat and at 298 K. Zr *K*-edge XAS spectra were acquired from 17990 to 18030 eV, resulting in a k-range up to 12 Å^{-1}.

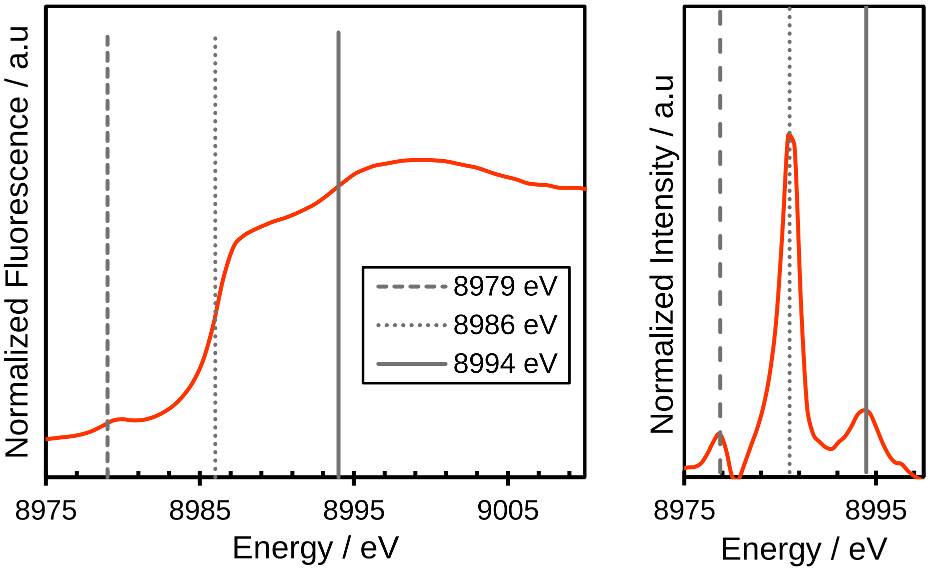

Supplementary Figure 17. Cu *K*-edge XANES spectrum of Cu-MOF-808 and first derivate analysis at 298 K.

The presence of a pre-edge signal (8986 eV) is consistent with a 1*s* to 4*p* transition + LMCT "shakedown" 3*p* of L to 3*d* of Cu^{2+}. Presence of a pre-edge signal (8979 eV) is consistent with a quadrupole allowed 1*s* to 3*d* transition or a distortion from a pure D_{4h} structure, square-planar to a D_{2d}, twisted square-planar. [6]

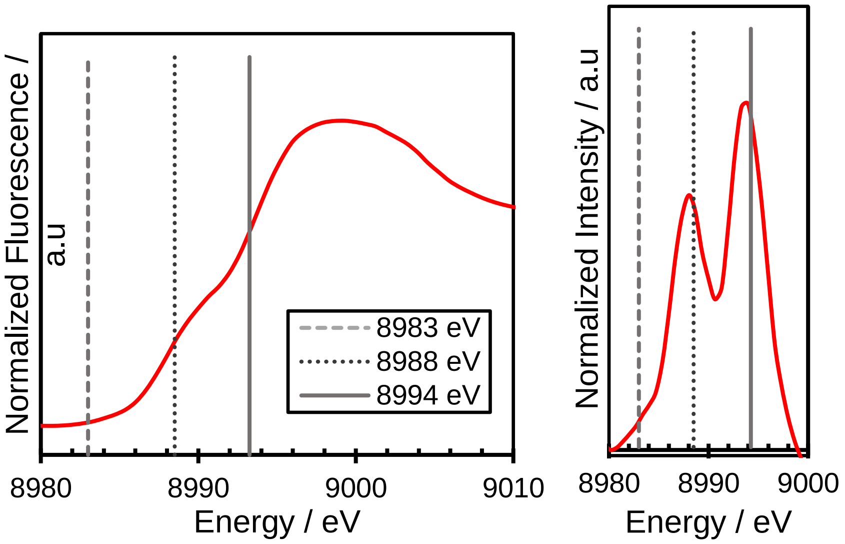

Supplementary Figure 18. Cu *K*-edge XANES spectrum of Cu-MOF-808 and first derivate analysis at 10 K.

For a higher resolution in the high energy EXAFS region, the experiments were conducted at 10 K. At this temperature the pre-edge region decreased in intensity dramatically, being therefore indicative of the proposed metal(3*d*)-Ligand(4*p*) orbital mixing. This mixing is directly associated with the vibronic structure of the system, which rapidly decreases with temperature.[7]

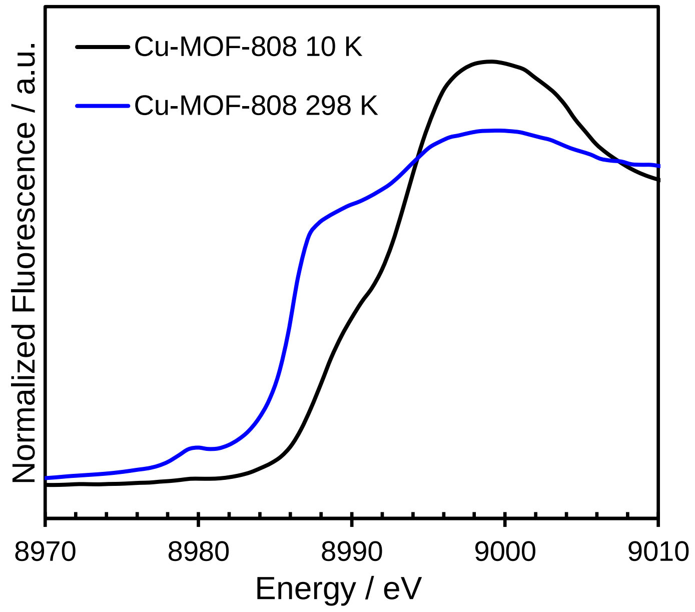

Supplementary Figure 19. Cu *K*-edge XANES data of Cu-MOF-808 at 10 K and at 298 K.

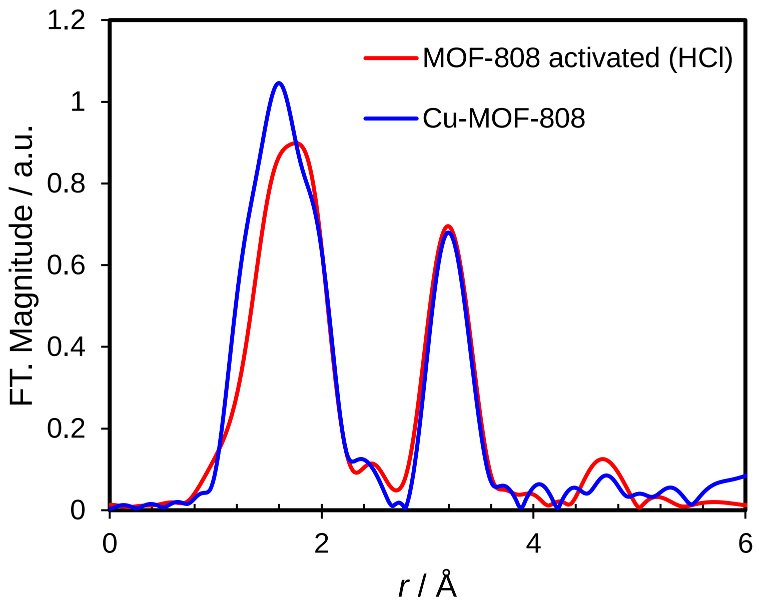

Supplementary Figure 20. Zr *K*-edge EXAFS data of MOF-808 and Cu-MOF-808.

Supplementary Note 10. X-ray photoelectron spectroscopy

X-ray photoelectron spectra were recorded with a lab-based spectrometer (SPECS GmbH, Berlin) using monochromated Al source (Al Kα_1 hν = 1486.6 eV) operated at 50W as excitation source. In the spectrometer, the X-ray is focused with a µ-FOCUS 600 monochromator onto a 300 µm spot on the sample, and the data is recorded with a PHOIBOS 150 NAP 1D-DLD analyser in fixed analyser transmission (FAT) mode. The pass energy was set to 40 eV for survey scans and 20 eV for high-resolution regions. The binding energy scale was calibrated using Au 4$f_{7/2}$ (84.01 eV) and Ag 3$d_{5/2}$ (368.20 eV). Charge compensation was required for data collection. Recorded spectra were additionally calibrated against the C 1s internal reference. *Data interpretation* was done with Casa XPS. Shirley or two-point linear background were used depending on the spectrum shape. Surface chemical analysis was done based on the peak area of high-resolution spectra and the CasaXPS sensitivity factors (where RSF of C 1*s* = 1.000).

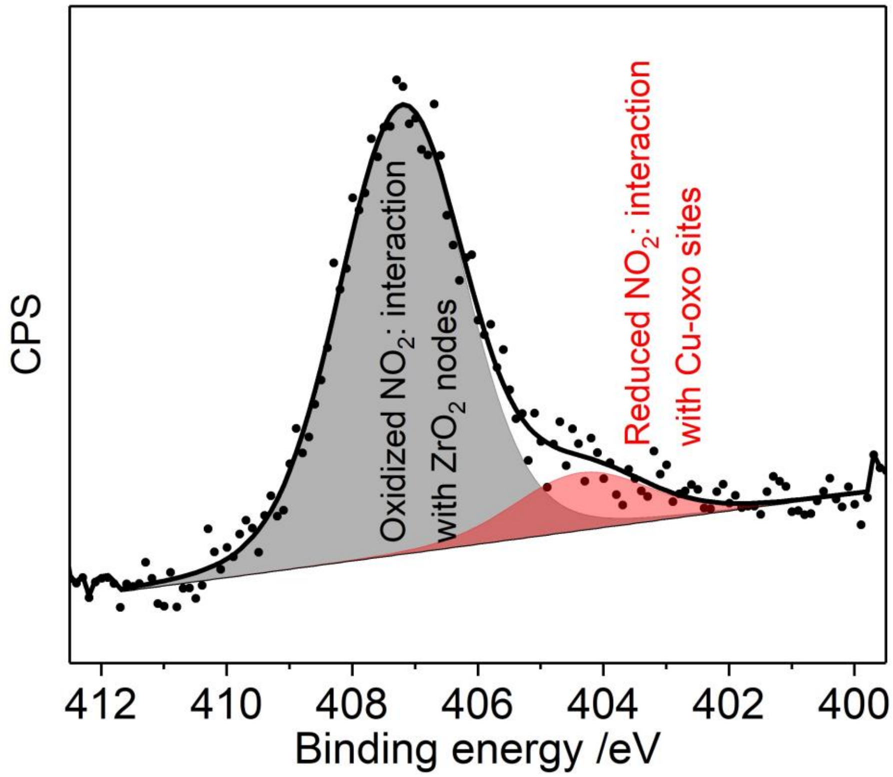

Supplementary Figure 21. XPS spectrum in the N1*s* for NO_2-loaded Cu-MOF-808, showing the presence of reduced chemisorbed NO_2 species.

Photoelectron spectra of Cu-MOF-808 in the Cu $2p_{3/2}$ region (Fig. S11.2). The spectrum is characteristic of Cu^{2+} (a d^9 cation), featuring a broad satellite next to the main peak due to localized final states (a), in which the valence hole remains mainly on the core-hole site, and a main peak (b), with most of the valence-hole density concentrated on the ligand sites surrounding the core-hole site.

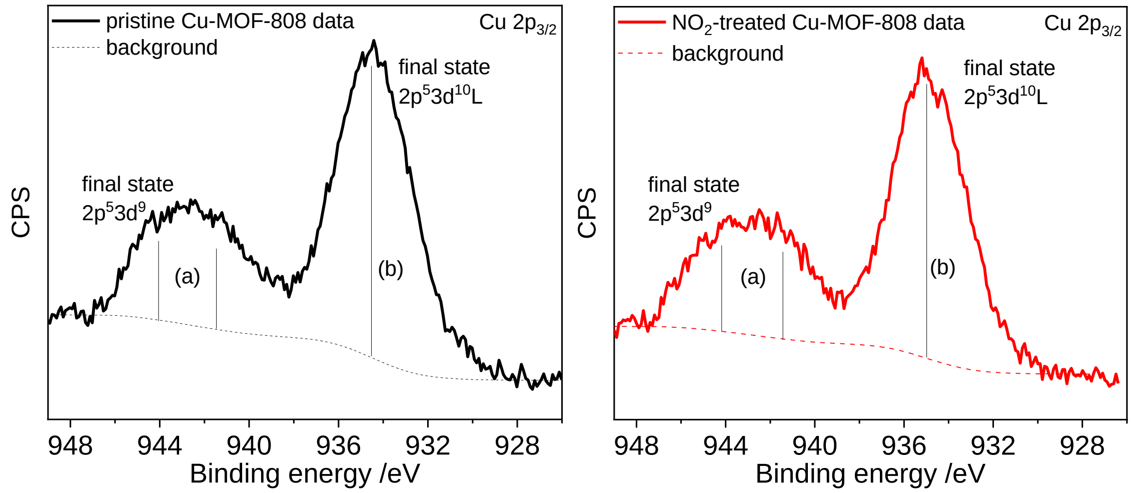

Supplementary Figure 22. XPS spectrum in the Cu 2*p*$_{3/2}$ for NO_2-loaded Cu-MOF-808, showing the presence of Cu(II) sites.

Photoelectron spectra of Cu-MOF-808 in the C 1*s* region (Fig. S11.3). Spectra before and after NO_2 exposure consists in two peaks that can be associated with aliphatic C species (at 285 eV) and C=O species (at 289 eV) in a ratio aliphatic:C=O 3:1. The absence of changes in the C 1*s* region demonstrates the robustness of Cu-MOF-808 towards the adsorption of NO_2.

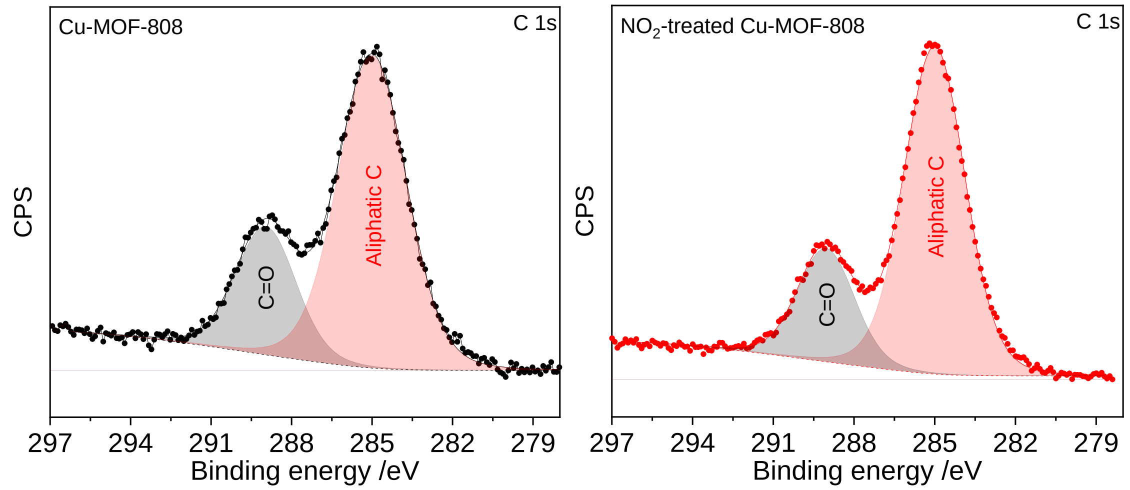

Supplementary Figure 23. XPS spectrum in the Cu 1*s* for NO_2-loaded Cu-MOF-808, showing the presence aliphatic and C=O species.

Supplementary Note 11. Computational methodology

Density Functional Theory (DFT) calculations were performed in order to elucidate the possible configurations of Cu-MOF-808. We modelled the structural and energetic properties of several mono- and bi-nuclear copper-oxo & copper-hydroxo clusters deposited on the nodes of the MOF-808. As a starting model for the pristine MOF-808, we choose a molecular cluster that is composed by two Zr_6O_8 octahedra bridged by 2 ligands. This model has been previously used in our previous work, where the deposition of iron-oxo clusters on the MOF-808 was investigated.[8] The coordinates of the starting model are carved from the experimentally determined crystal structure. The benzene-tricarboxylate ligands, which are bridging the two Zr_6O_8 octahedra, are cropped to benzene-dicarboxylate. The remaining four ligands are also cropped to formate. Based on the experimental observations, six (6) formate molecules are further added as capping ligands. For charge balancing, four (4) protons have to be added to the μ_3-O atoms of each Zr_6O_8 octahedron. As a next step, four formate capping ligands are removed, with each one being replaced by a hydroxo and a water molecule, giving rise to the MOF-808 model, where the copper-hydroxide species will be deposited.

Subsequently, we investigated the structural and energetic characteristics of the deposition of two Cu(II) atoms on the nodes of the MOF-808 model. Two possible ways of deposition have been considered: i) two mono-nuclear, and ii) one bi-nuclear copper-oxo & copper-hydroxo clusters. To reduce the complexity of the system, due to many possible combinations to couple the unpaired electrons of the Cu(II)/Cu(II) atoms, we decided to study only the deposition of the high-spin ferromagnetically coupled Cu(II)-Cu(II) pairs with a spin multiplicity of 3. Because of the different stoichiometries of the resulting structures, the comparison is done by computing the formation energies of the $Cu_2O_x(OH)_y(H_2O)_z$ with the equation: ΔE^{form} = E(Cu-MOF-808) -E(MOF-808) +mE(H_2O) - E(MOF-808) - nE(precursor), where E are the energies of the Cu-MOF-808, MOF-808, H_2O, and the copper precursor molecules, and m, n are the number of water and precursor molecules in the formation reaction respectively. As Cu(II) precursor, a molecule with the stoichiometry $Cu(OH)_2(H2O)_2$ is considered.

As a final step, after obtaining the most stable configurations for the deposited Cu(II) atoms, the interactions with NO_2 have been computed. Several configurations have been considered and the interaction energies with NO_2 are computed with the equation: I.E = E(NO_2-Cu-MOF-808) – E(Cu-MOF-808) - E(NO_2), where E are the energies of the NO_2 complex with Cu-MOF-808, Cu-MOF-808 and NO_2, respectively. The total spin multiplicity of the complexes is considered to be a quartet.

During all geometry optimizations, some restrictions have to be applied in order to mimic the crystal environment. Two (2) of the zirconium atoms at the edges of the molecular cluster, and twenty-four (24) oxygen atoms that belong to the ligands are kept frozen. The r2-SCAN-3c functional in combination with the def2-mTZVPP basis set have been used for all geometry optimizations. This low-cost density functional has been shown to perform very well for open-shell transition metal reactions.[9, 10] Finally, single point energies with the M06-L functional in combination with def2-TZVPP have been performed at the r2-SCAN-3c optimized geometries.[11] All calculations have been performed using the ORCA 5.0.3 program.[12]

Supplementary Note 12. Computational results

Due to the rich proton topology of MOF-808, several possibilities exist, how a $Cu_2O_x(OH)_y(H_2O)_z$ cluster can be deposited on the nodes of the MOF after reaction of a copper precursor with the available protons from the μ_3-OH, terminal-OH and terminal-Aqua ligands. Moreover, the copper species could be deposited either as two isolated, or as bridged-(hydr)oxo. Initially, we investigate, how the first copper can be attached. This can be done either through the reaction of the precursor with the terminal oxygen atoms or with the μ_3. The calculations show that adsorption is more favorable, when the copper atom interacts through the μ_3- and terminal-oxygen atoms than with the two terminal-oxygen atoms only. The two adsorption schemes are denoted as (μ_3-OZr/t-OZr)-Cu(OH)(H_2O) and (t-OZr/t-OZr)-Cu(OH)(H_2O) respectively and are shown in Supplementary Figure 24. The relative energy difference between these two adsorption modes is ~48 and ~29 kJ/mol according to the r2-SCAN-3C/def2-mTZVPP and M06L/def2-TZVPP methods. In the case of adsorption on the terminal-oxygen atoms, the copper atom has one terminal hydroxo and one aqua ligand, which interact via hydrogen bonding with the terminal-hydroxo and aqua ligands of another zirconia node. Subsequently, based on the observation for the deposition of one copper atom, several possible configurations have been considered for the deposition of two copper atoms. Similarly, the deposition as two (μ_3-OZr/t-OZr)-Cu(OH)(H_2O) clusters (model A-2Cu) is energetically favored by ~41 (~35) kJ/mol with respect to two (t-OZr/t-OZr)-Cu(OH)(H_2O) (model C-2Cu) according to the r2-SCAN-3c (M06L). All configurations are presented in the Supplementary Figure 24 and Supplementary Table 4.

Supplementary Table 4: Formation energies (in kJ/mol) of a $Cu_{1,2}O_x(OH)_y(H_2O)_z$ clusters deposited on the MOF-808 nodes calculated with the r2-SCAN-3c/def2-mTZVPP and M06-L/def2-TZVPP. The stoichiometries of the reaction energies, important distances (Cu-Cu, Cu-Zr in Å) and the absorption manner (via the terminal- or the μ_3-O) are also reported.

Model	*ΔE^{form} (kJ/mol)*		*Reaction*	*Absorption mode of Cu*	*R(Cu-Cu) / R(Cu-Zr), Å*
	r2-SCAN-3c	*M06-L*			
A-1Cu	-116.3	-93.3	MOF + 1Cu-prec → M01-1Cu + 2H_2O	(μ_3-O, t-O)	-- / 3.01
B-1Cu	-68.7	-63.9	MOF + 1Cu-prec → M02-1Cu + 2H_2O	(t-O, t-O)	-- / 3.15
C-1Cu	-74.8	-71.3	MOF + 1Cu-prec → M03-1Cu + 2H_2O	(μ_3-O, t-O)	-- / 3.39
D-1Cu	56.3	71.7	MOF + 1Cu-prec → M04-1Cu + 2H_2O	t-O	-- / 3.24
E-1Cu	95.9	101.1	MOF + 1Cu-prec → M05-1Cu + 2H_2O	t-O	-- / 3.26
A-2Cu	-248.8	-204.2	MOF + 2Cu-prec → A + 4H_2O	(μ_3-O, t-O)	-- / 3.01 & 3.01
B-2Cu	-208.3	-165.2	MOF + 2Cu-prec → B + 4H_2O	(t-O, t-O)	4.13 / 3.28 & 3.31
C-2Cu	-213.9	-190.2	MOF + 2Cu-prec → C + 4H_2O	(t-O, t-O)	3.35 / 3.25 & 3.25
D-2Cu	-185.0	-165.9	MOF + 2Cu-prec → D + 4H_2O	(t-O, t-O)	3.69 / 3.23 & 3.51
E-2Cu	-168.4	-138.7	MOF + 2Cu-prec → E + 4H_2O	(t-O, t-O)	3.29 / 3.15 & 3.34
F-2Cu	-140.7	-126.7	MOF + 2Cu-prec → F + 4H_2O	(t-O, t-O)	3.65 / 3.28 & 3.42

G-2Cu	-21.3	8.5	MOF + 2Cu-prec → G + $6H_2O$	(t-O, t-O)	3.33 / 3.07 & 3.19
H-2Cu	12.3	44.5	MOF + 2Cu-prec → H + $6H_2O$	(t-O, t-O)	3.18 / 3.27 & 3.25
I-2Cu	15.1		MOF + 2Cu-prec → I + $6H_2O$	(t-O, t-O)	3.09 / 3.27 & 3.30
J-2Cu	151.6	183.7	MOF + 2Cu-prec → J + $6H_2O$	(t-O, t-O)	2.64 / 3.00 & 3.00

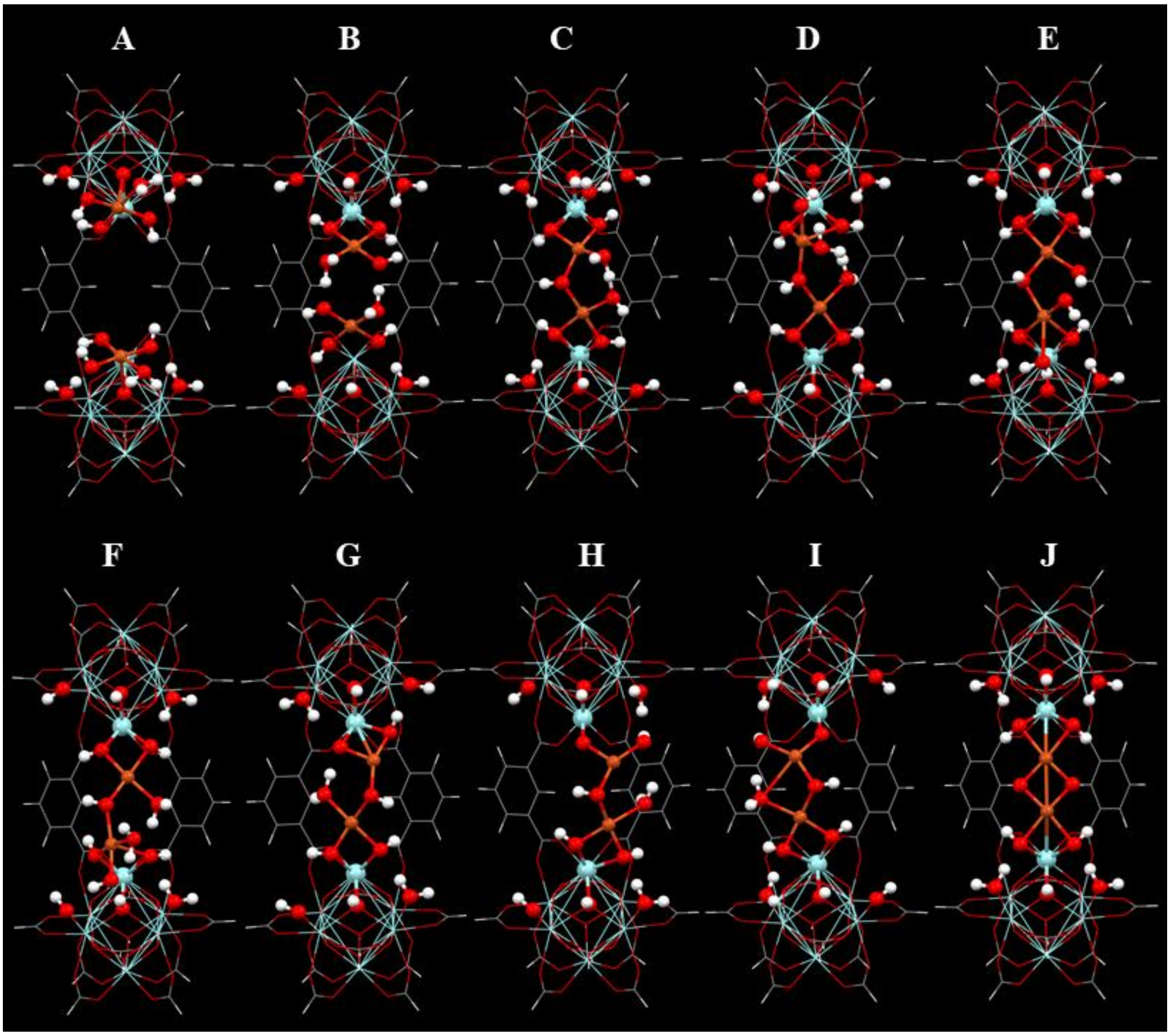

Supplementary Figure 24. (A-J) Geometries of all computed model with two copper atoms deposited on MOF-808. See Supplementary Table 4 for their energetics.

We have also calculated the interaction energies between NO_2 and the three most stable models of the Cu-MOF-808. For comparison reasons, we have also considered the adsorption of NO_2 on the MOF-808 and the Cu-HKUST-1, which is a MOF containing unsaturated copper metal sites in a similar distorted square planar environment. The results are summarized in Supplementary Table 5 and Supplementary Figure 25 .

Supplementary Table 5: Interaction energies (in kJ/mol) of NO_2 with the three most stable models the Cu-MOF-808, the MOF-808 and the Cu-HKUST-1. Interaction energies are computed from the r2-SCAN-3c/def2-mTZVPP method. The stoichiometries of the reaction energies, important distances (Cu-Cu, Cu-Zr in Å) and the absorption manner (via the terminal- or the μ_3-O) are also reported.

MOF	*Model*	*I.E (kJ/mol)*	*Binding mode*
		r2-SCAN-3c/def2-mTZVPP	
Cu-MOF-808	A-2Cu	-64.3	Cu…NO_2
		-41.3	Cu..ONO
	B-2Cu	-38.3	Cu…NO_2
		-36.6	Cu..ONO
	C-2Cu	-59.1 / -48.5	Cu…NO_2
		-55.2 / -31.2	Cu..ONO
MOF-808	(1)	-22.5	
	(2)	-22.1	
	(3)	-28.6	
HKUST-1		-27.9	Cu…NO_2
		-32.2	Cu..ONO

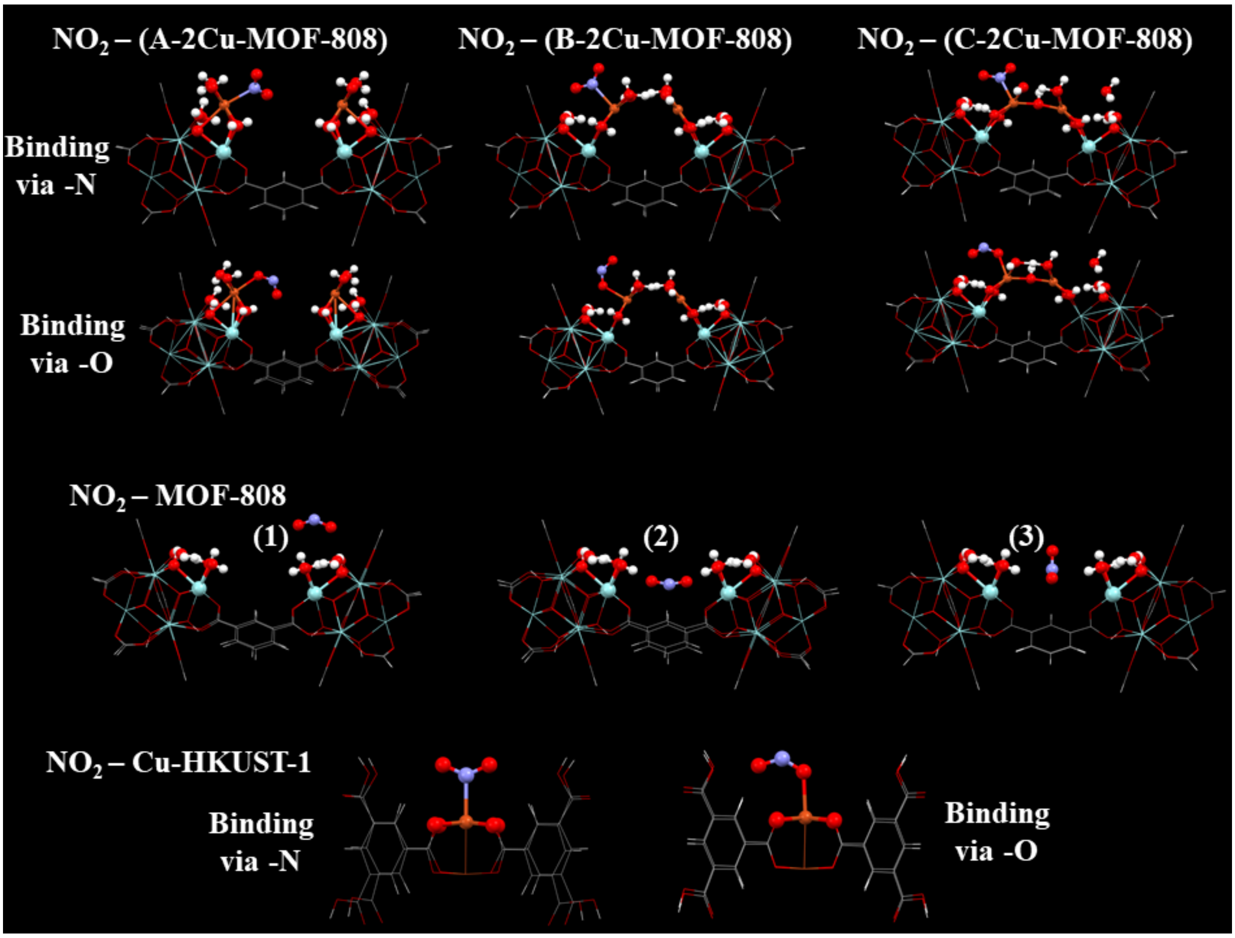

Supplementary Figure 25. Optimized geometries of NO_2 interacting with the Cu-MOF-808, MOF-808 and Cu-HKUST-1 frameworks.

In Cu-MOF-808, the most stable adsorption geometry of NO_2 on the copper site is via its nitrogen atom. Interestingly, the most stable adsorption mode is via the oxygen atom of the

NO_2 with the copper atom in the HKUST-1. This can be justified by the presence of additional dispersive interactions of the NO_2 with the hydroxo groups of the MOF-808. This type of interaction is not present in the HKUST-1, where the NO_2 interacts only with the copper atom.

The two types of interaction are qualitatively illustrated by performing an Interaction Region Indicator (IRI) analysis and plotting the results in the Supplementary Figure 26. The IRI plots indicate vdW interactions between the NO_2 and the μ_3-OH groups as illustrated with isosurfaces of green colour in part A of Fig S.12.3. Stronger interactions between NO_2 and the copper sites are demonstrated by the isosurfaces of dark blue colour in part A of the Supplementary Figure 26, while simultaneously areas dominated by weaker vdW interactions exist as evidenced by the green isosurfaces.

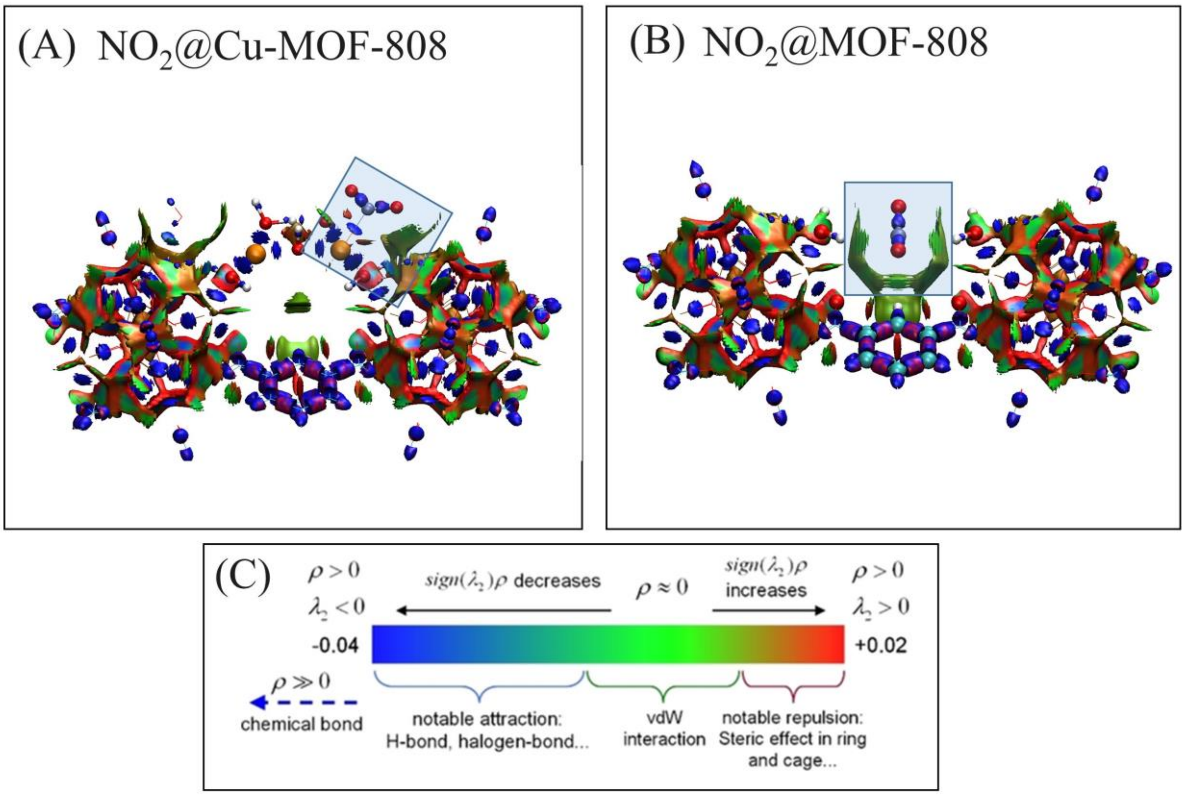

Supplementary Figure 26. Interaction Region Indicator (IRI) Plots of the: A) NO_2 interacting with the Cu-MOF-808, B) NO_2 interacting with the MOF-808. C) Standard coloring method and chemical explanation of the $sign(\lambda_2)\rho$ on IRI isosurfaces

Competitive adsorption of other common gases in the Cu-MOF-808:

We have also explored computationally the nature of the binding between competing molecules (such as H_2O, NO, CO and CO_2) with the Cu-MOF-808. The relative strength of the interactions between the three competing molecules (NO_2, NO, CO_2, CO and H_2O) and the copper site is used as a qualitative descriptor to predict, which of the above molecules will be preferentially adsorbed. However, recent experimental and computational studies[13,14] have shown that the binding strength does not always indicate the preferred adsorbed molecule, and that a combination/competition between thermodynamics (binding energies) and kinetics (energy barriers) defines the adsorbed molecule. For example, although NH_3 has stronger binding than H_2O with the open metal sites of the Ni- and Mg-MOF-74, the

presence of multiple H_2O molecules will cause the displacement of a preabsorbed NH_3 molecule.[13] Moreover, the displacement of CO bound inside Ni-MOF-74 (binding energy of 53 kJ/mol) is readily driven by CO_2 exposure, even though CO_2 has a noticeably weaker binding energy of 41 kJ/mol.[14]

Here, we have attempted to assess the competitive adsorption of NO_2, NO, CO_2, CO and H_2O by comparing their interaction energies with the copper sites. The results are presented in the Supplementary Table 6. The copper sites have distorted square-planar geometries. Therefore, the binding site of the adsorbed molecule is considered to be on the axial position.

Supplementary Table 6: Interaction energies (in kJ mol^{-1}) and distances (in Å) of NO_2, NO, CO_2 and H_2O interacting with the copper sites of model-C-2Cu.

model-C-2Cu	I.E. (kJ mol^{-1})[a]	Charge Transfer Cu-MOF-800 → molecule (AIM)[b]	I.E. (kJ mol^{-1})[a]	Charge Transfer Cu-MOF-800 → molecule (AIM)[b]
NO_2	-59.1	+0.23	-48.5	+0.16
NO	-43.6	+0.01	-38.3	+0.03
CO_2	-50.0	+0.01	-41.2	+0.01
CO	-44.8	-0.01	-39.5	+0.01
H_2O	-66.9 (first[c])<br>-66.3 (second[c])<br>-73.1 (third[c])	n.c	-77.3	0.00

[a] Two values for the interaction energies are reported, because two copper sites with slightly different coordination environment are present.
[b] Positive value for the computed charge transfer means that charge is flowing from the MOF towards the adsorbed molecule.
[c] The interaction of up to three H_2O molecules with the copper site has been investigated in this case. In all of these three cases, the water molecule moves away from the copper site and interacts with hydrogen-bonds with the neighbouring hydroxo and aqua ligands of the ZrO_2 node.

In the cases of NO and CO, the interaction energies with the Cu(II) sites are weaker than the interaction of NO_2. This is consistent with previous works by us[15] and others[16] on the CO and NO_x binding with the open metal sites of the HKUST-1 MOF. This is not unexpected, considering that CO and NO interact stronger with Cu(I) sites than the Cu(II) due to significant pi-backdonation.[17] The interaction energies of CO_2 with the two Cu(II) sites are computed weaker compared to NO_2, with values of -50.0 and -41.2 kJ mol^{-1}. Unexpectedly, the preferred adsorption site of the water molecule is not on the copper site. During the geometry optimization, the water molecule moves from the axial site of the Cu(II) towards the ZrO_2 node and prefers to interact via Hydrogen-bonds with the hydroxo and aqua ligands. Same results are obtained, when a second and third water molecule are inserted on top of the copper site. In all cases, the water molecules move away from the axial binding site of the Cu(II). Although, the interaction energies of H_2O with the Cu(II) sites are calculated to be stronger than of NO_2, the presence of water molecule will not affect the sensing, because the axial site of one copper centre is still available for binding with NO_2 in the presence of water molecules. In the second copper site, the water molecule remains adsorbed on the metal axial position with an interaction energy of -77.3 kJ mol^{-1} that is significantly stronger than the computed interaction energy of NO_2 (-48.5 kJ mol^{-1}). Thus, at humid conditions one of the two copper sites will be still available for adsorbing and sensing NO_2.

In conclusion, the calculations suggest that NO, CO and CO_2 are adsorbed less strongly than the NO_2, and that at humid conditions one of the two copper sites will be able to bind NO_2.

Supplementary Note 13. Optical sensing

Room temperature photoluminescence measurements in the presence of NO_2 (50 ppm NO_2 in synthetic air) were performed by enclosing the MOF in a home-made gas chamber with optical access. The MOF powder was loaded in the recess of an aluminum plate covered by a hollow copper grid. The gas flow was controlled with flow meters. The MOF powders were photoexcited by a TEEM Photonics Nd:YAG laser (λ=355 nm), delivering pulses of 300 ps duration at repetition rates from single shot to 1 kHz. Photoluminescence was free-space collected at approximately 20° respect to the incident beam by an Acton Research SP2500 spectrometer (f= 500 mm) equipped with a Princeton Instruments Spec-10 liquid nitrogen cooled back-illuminated deeply depleted CCD for the acquisition of PL spectra. The scattered light arising from the excitation line was appropriately cut by placing a 370 nm long-pass filter in front of the spectrometer.

Supplementary Figure 27 presents the activation process of Cu-MOF-808 by purging N_2 for an hour. As it can be seen, the activation takes to exploit in gas sensing measurements. (All the measurements for Cu-MOF-808 were performed after the activation)

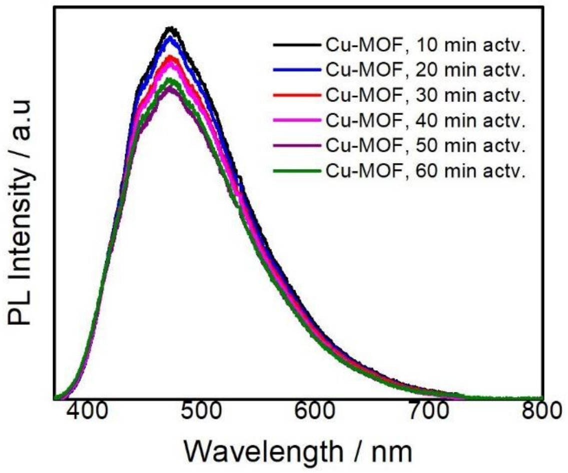

Supplementary Figure 27. PL spectra of Cu-MOF-808 upon exposure to N_2 gas for activation (λ_{exc}: 355 nm).

In order to know how the results are comparative, sensing measurements were performed for MOF-808. Similarly, the MOF-808 powder was activated by purging N_2 gas for 20 min. According to Supplementary Figure 28, the activation time for MOF-808 is half of the Cu-MOF-808.

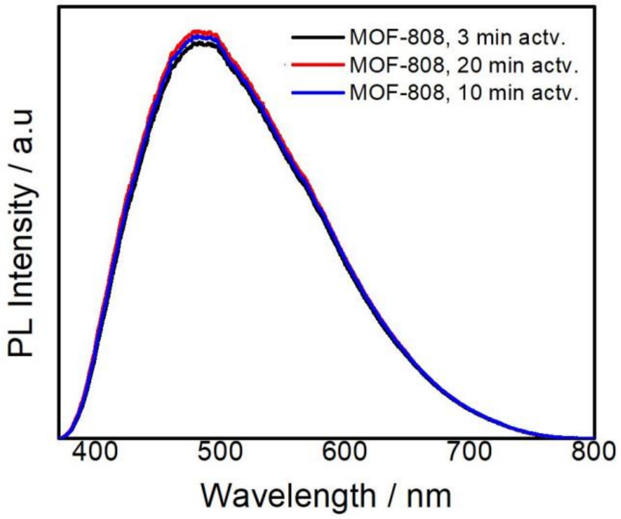

Supplementary Figure 28. PL spectra of MOF-808 upon exposure to N_2 gas for activation (λ_{exc}: 355 nm).

Supplementary Figure 29 shows the sensing response of Cu-MOF-808 after nine NO_2–N_2 cycles (approximately an hour of operation), demonstrating that Cu-MOF-808 is a promising and efficient material for NO_2 sensing. In the legend, the times represent the time exposure of Cu-MOF-808 to NO_2 (50 ppm) and N_2 after approximately an hour.

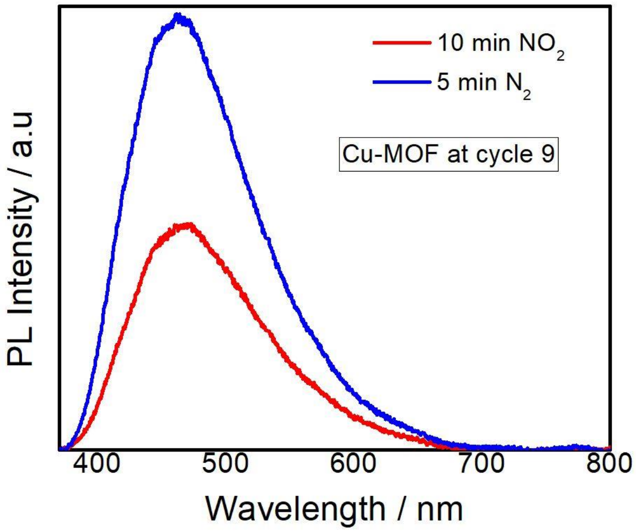

Supplementary Figure 29. PL spectra of Cu-MOF-808 upon exposure to the gases at cycle 9 (λ_{exc}: 355 nm).

Supplementary Figure 30 depicts a similar measurement on less responsive MOF-808. Despite its fast gas uptake that is related to its higher porosity with respect to Cu-MOF-808, its sensing efficiency decreases noticeably after an hour.

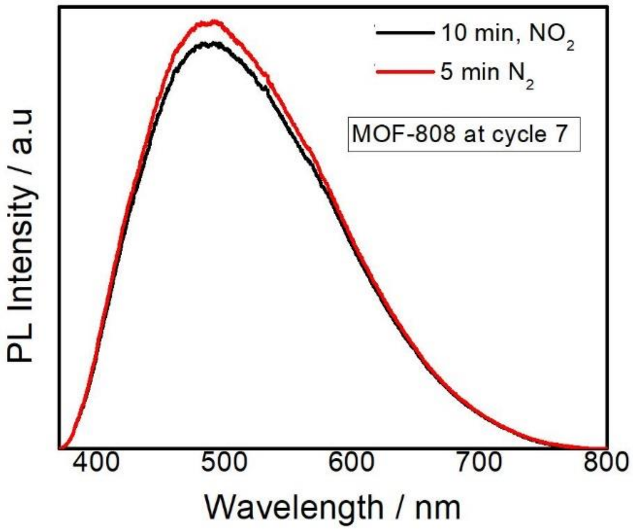

Supplementary Figure 30. PL spectra of MOF-808 upon exposure to the gases at cycle 7 (λ_{exc}: 355 nm).

And Supplementary Figure 31 depicts the PL spectra of MOF-808 and Cu-MOF-808 in powder after activating with N_2 gas in turn for 20 and, 60 min. Incorporating Cu (II) ions to MOF-808 led to 10 nm blue-shift in emission spectrum.

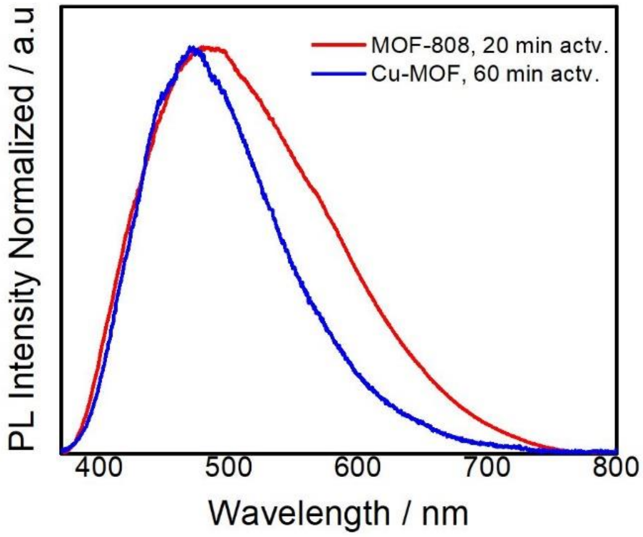

Supplementary Figure 31. PL spectra of MOF-808 and Cu-MOF-808 after activating with N_2 (λ_{exc}: 355 nm).

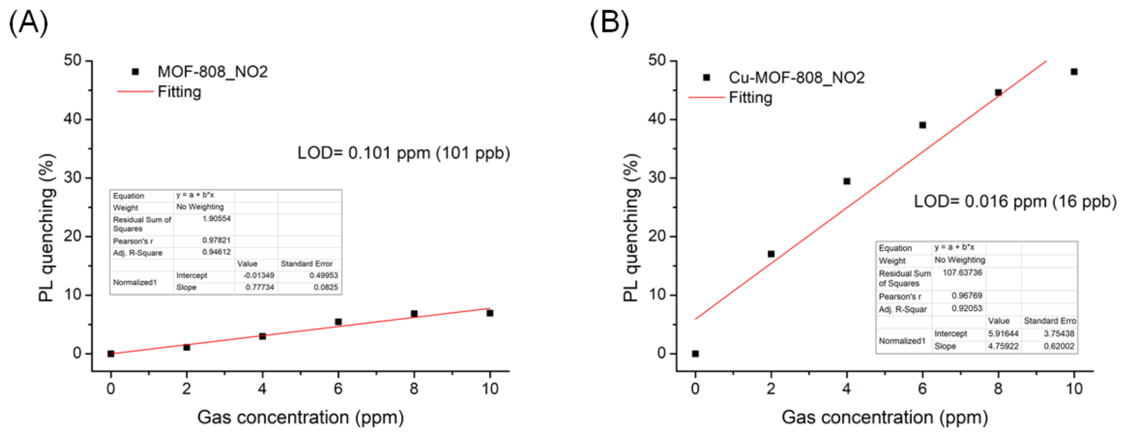

Supplementary Figure 32. Linear fitting the PL quenching of (A) MOF-808 and (B) Cu-MOF-808 exposed to different concentrations of NO_2 (0 – 10 ppm).

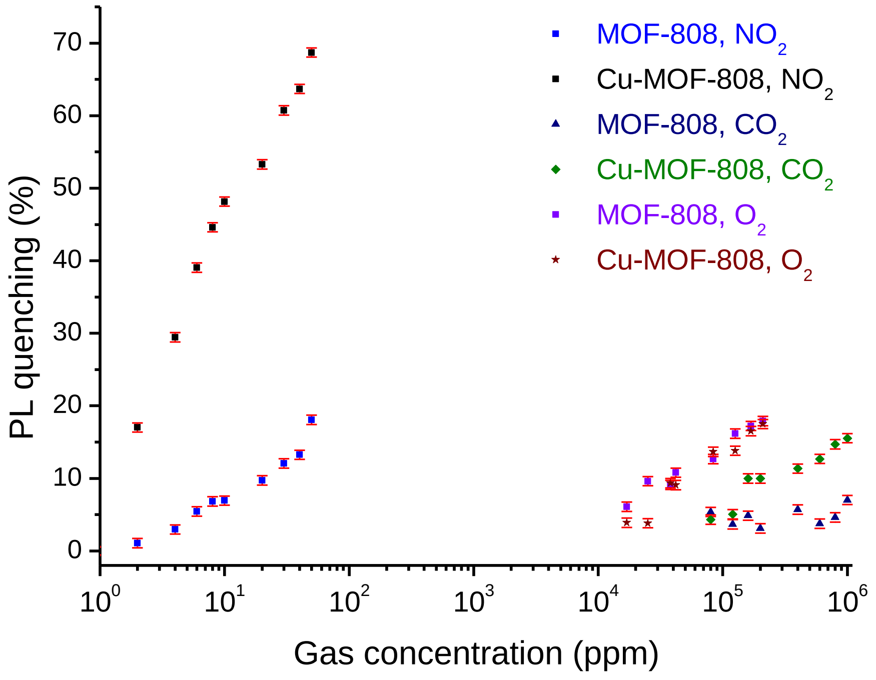

Supplementary Figure 33. PL quenching of MOF-808 and Cu-MOF-808 exposed to different concentrations of NO_2, CO_2 and O_2 after activating with N_2 (λ_{exc}: 355 nm).

Comparative analyses

Supplementary Table 7: Comparison of different materials employed as NO_2 sensors reported in the literature.

Material	Sensor type	T (ºC)	Concentration, LOD	Response	Ref.
In/ZnO-10	Chemoresistive	300	0.20 ppb	Electrical resistance	[18]
[Ni(TPyP)-(TiF$_6$)]@PDVT-10	OFET/ Chemoresistive	25	8.25 ppb	Electrical resistance	[19]
Co_3O_4/Biomass carbon	Chemoresistive	25	10 ppb	Electrical conductivity	[20]
In_2O_3/ZIF-8	Chemoresistive	140	10 ppb	Electrical resistance	[21]
$Cu_3(HHTP)_2/Fe_2O_3$	Chemoresistive	20	11 ppb	Electrical resistance	[22]
CuO tube-like nanofibers	Chemoresistive	25	12.2 ppb	Electrical resistance	[23]
PDVT-10	OFET /Chemoresistive	25	25 ppb	Electrical resistance	[24]
Ti-FIR-120	Chemoresistive	25	40 ppb	Electrical resistance	[25]
Pt@$Cu_3(HHTP)_2$ thin-film	Chemoresistive	25	0.1 ppm	Electrical resistance	[26]
$Cu_3(HHTP)_2$ thin-film	Chemoresistive	25	0.1 ppm	Electrical resistance	[26]
Cu-MOF250 (unknown structure)	Chemoresistive	40	0.14 ppm	Electrical resistance	[27]
Ag-3 doped-WO_3	Chemoresistive	30	196.8 ppb	Electrical resistance	[28]
Ni-MOF-74	Capacitive	50	0.5 ppm	Electrical resistance	[29]
Pd@$Cu_3(HHTP)_2$ thin-film	Chemoresistive	25	1 ppm	Electrical resistance	[30]
Pt@$Cu_3(HHTP)_2$ thin-film	Chemoresistive	25	1 ppm	Electrical resistance	[30]
$NiFe_2O_4$ nanofibers	Chemoresistive	350	10 ppm	Electrical resistance	[31]
$((H_3O)_4[Co_2(L)(DMF)(H_2O)_4]_2 DMF\cdot 3H_2O)$	Chemoresistive	25	-	Electrical conductivity	[32]
ZIF-8	Chemoresistive	350	-	Electrical resistance	[33]
Porphyrin LB Films	Optical	25	0.46 ppm	Red-shift of UV-Vis spectrum	[34]
TCPC@MOF@PDMS	Optical	25	> 0.5 ppm	PL quenching	[35]
Porphyrin-film@SiO_2	Optical	25	1.0 ppm	UV-Vis peaks shift/Intensity change and turn-on absorption	[36]
$\{[Tb_2(NBDC)_3(DMF)_4]2DMF\}$	Optical	25	1.8 ppm	PL quenching	[37]
$\{[Eu_2(NBDC)_3(DMF)_4]2DMF\}$	Optical	25	2.2 ppm	PL enhancement	[37]
Tb(BTC)@PDMS	Optical	25	4 ppm	PL quenching	[38]
Calixarene-based Zr-MOF film	Optical/ Chemoresistive	25	5 ppm	Colorimetric/ Turn-on UV-vis absorption/photo-current	[39]

				change	
Zn-ZJU-66 film	Optical	25	-	PL quenching	40
Y-DOBDC	Optical	25	-	Colorimetric/ PL quenching	41
$((H_3O)_4[Co_2(L)(DMF)(H_2O)_4]_2$ $DMF\cdot 3H_2O)$	Optical/ Chemoresistive	25	-	Colorimetric/ Conductivity	32
**MOF-808**	**Optical**	**25**	**101 ppb**	**PL quenching**	**In this work**
**Cu- MOF-808**	**Optical**	**25**	**16 ppb**	**PL quenching**	**In this work**

Supplementary References

(1) Furukawa, H.; Gándara, F.; Zhang, Y. B.; Jiang, J.; Queen, W. L.; Hudson, M. R.; Yaghi, O. M. Water Adsorption in Porous Metal-Organic Frameworks and Related Materials. *J. Am. Chem. Soc.* **136** (11), 4369–4381 (2014).

(2) Romero-Muñiz, I.; Romero-Muñiz, C.; del Castillo-Velilla, I.; Marini, C.; Calero, S.; Zamora, F.; Platero-Prats, A. E. Revisiting Vibrational Spectroscopy to Tackle the Chemistry of Zr_6O_8 Metal-Organic Framework Nodes. *ACS Appl. Mater. Interfaces* **14**, 23, 27040–27047 (2022).

(3) Basham, M.; Filik, J.; Wharmby, M. T.; Chang, P. C. Y.; el Kassaby, B.; Gerring, M.; Aishima, J.; Levik, K.; Pulford, B. C. A.; Sikharulidze, I.; Sneddon, D.; Webber, M.; Dhesi, S. S.; Maccherozzi, F.; Svensson, O.; Brockhauser, S.; Náray, G.; Ashton, A. W. Data Analysis WorkbeNch (DAWN). *J Synchrotron Radiat*. **22**, 853–858 (2015).

(4) Juhás, P.; Davis, T.; Farrow, C. L.; Billinge, S. J. L. PDFgetX3: A Rapid and Highly Automatable Program for Processing Powder Diffraction Data into Total Scattering Pair Distribution Functions. *J. Appl. Crystallogr.* **46** (2), 560–566 (2013).

(5) Ravel, B.; Newville, M. ATHENA, ARTEMIS, HEPHAESTUS: Data Analysis for X-Ray Absorption Spectroscopy Using IFEFFIT. *J. Synchrotron Radiat.* **12** (4), 537–541 (2005).

(6) Baker, M. L.; Mara, M. W.; Yan, J. J.; Hodgson, K. O.; Hedman, B.; Solomon, E. I. K- and L-Edge X-Ray Absorption Spectroscopy (XAS) and Resonant Inelastic X-Ray Scattering (RIXS) Determination of Differential Orbital Covalency (DOC) of Transition Metal Sites. *Coord. Chem. Rev.* **345**, 182–208 (2017).

(7) Shulman, G. R.; Yafet, Y.; Eisenberger, P.; Blumberg, W. E. Observations and Interpretation of X-Ray Absorption Edges in Iron Compounds and Proteins. *Proc. Natl. Acad. Sci. USA* **73** (5), 1384–1388 (1976).

(8) Castillo-Blas, C.; Romero-Muñiz, I.; Mavrandonakis, A.; Simonelli, L.; Platero-Prats, A. E. Unravelling the Local Structure of Catalytic Fe-Oxo Clusters Stabilized on the MOF-808 Metal Organic-Framework. *Chem. Comm.* **56** (100), 15615–15618, (2020).

(9) Grimme, S.; Hansen, A.; Ehlert, S. R2SCAN-3c: A “Swiss Army Knife” Composite Electronic-Structure Method. *J. Chem. Phys.* **154**, 64103 (2021).

(10) Maurer, L. R.; Bursch, M.; Grimme, S.; Hansen, A. Assessing Density Functional Theory for Chemically Relevant Open-Shell Transition Metal Reactions. *J. Chem. Theory Comput*. **17,** 6151 (2021).

(11) Zhao, Y.; Truhlar, D. G. A New Local Density Functional for Main-Group Thermochemistry, Transition Metal Bonding, Thermochemical Kinetics, and Noncovalent Interactions. *J. Chem. Phys*. **125**, 194101 (2006).

(12) Neese, F. Software Update: The ORCA Program System—Version 5.0. *Comput. Mol. Sci.* **12**, 5 (2022).

(13) Tan, K.; Ullah, S.; Pandey, H.; Cedeñ O-Morales, E. M.; Wang, H.; Wang, K.; Zhou, H.-C.; Li, J.; Thonhauser, T. Competitive Adsorption of NH3 and H2O in Metal−Organic Framework Materials: MOF-74. *Chem. Mater*. **34**, 17, 7906–7915 (2022).

(14) Pandey, H.; Wang, H.; Feng, L.; Wang, K.-Y.; Zhou, H.-C.; Li, J.; Thonhauser, T.; Tan, K. Revisiting Competitive Adsorption of Small Molecules in the Metal−Organic Framework Ni-MOF-74. *Inorg. Chem*. **62**, 950–956 (2023).

(15) Supronowicz, B.; Mavrandonakis, A.; Heine, T. Interaction of Small Gases with the Unsaturated Metal Centers of the HKUST-1 Metal Organic Framework. *J. Phys. Chem. C* **117,** 28, 14570–14578 (2013).

(16) Zong, S.; Zhang, Y.; Lu, N.; Ma, P.; Wang, J.; Shi, X. R. A DFT Screening of M-HKUST-1 MOFs for Nitrogen-Containing Compounds Adsorption. *Nanomaterials* **8**, 958 (2018).

(17) Drenchev, N.; Rosnes, M. H.; C Dietzel, P. D.; Albinati, A.; Hadjiivanov, K.; Georgiev, P. A. Open Metal Sites in the Metal−Organic Framework CPO-27-Cu: Detection of Regular and Defect Copper Species by CO and NO Probe Molecules. *J. Phys. Chem. C*, **122**, 30, 17238–17249 (2018).

(18) Li, Z.; Zhang, Y.; Zhang, H.; Jiang, Y.; Yi, J. Superior NO_2 Sensing of MOF-Derived Indium-Doped ZnO Porous Hollow Cages. *ACS Appl. Mater. Interfaces* **12**, 37489–37498 (2020).

(19) Yuvaraja, S.; Surya, S. G.; Chernikova, V.; Teja Vijjapu, M.; Shekhah, O.; Bhatt, P. M.; Chandra, S.; Eddaoudi, M.; Salama, K. N. Realization of an Ultrasensitive and Highly Selective OFET NO_2 Sensor: The Synergistic Combination of PDVT-10 Polymer and Porphyrin−MOF. *ACS Appl. Mater. Interfaces* **12,** 16, 18748–18760 (2020).

(20) Chen, J.; Lv, H.; Bai, X.; Liu, Z.; He, L.; Wang, J.; Zhang, Y.; Sun, B.; Kan, K.; Shi, K. Synthesis of Hierarchically Porous Co3O4/Biomass Carbon Composites Derived from MOFs and Their Highly NO2 Gas Sensing Performance. *Microporous and Mesoporous Mater.* **321**, 111108 (2021).

(21) Liu, Y.; Wang, R.; Zhang, T.; Liu, S.; Fei, T. Zeolitic Imidazolate Framework-8 (ZIF-8)-Coated In_2O_3 Nanofibers as an Efficient Sensing Material for Ppb-Level NO_2 Detection. *J. Colloid Interface Sci*. **541**, 249–257 (2019).

(22) Jo, Y.-M.; Lim, K.; Won Yoon, J.; Kun Jo, Y.; Kook Moon, Y.; Won Jang, H.; Lee, J.-H. Visible-Light-Activated Type II Heterojunction in Cu_3(Hexahydroxytriphenylene)$_2$/Fe_2O_3 Hybrids for Reversible NO_2 Sensing: Critical Role of Π−π* Transition. *ACS Cent. Sci.* **7**, 1176–1182 (2021).

(23) Liu, J.; Wang, W.; Li, G.; Bian, X.; Liu, Y.; Zhang, J.; Gao, J.; Wang, C.; Zhu, B.; Lu, H. Metal–Organic Framework-Derived CuO Tube-like Nanofibers with High Surface Area and Abundant Porosities for Enhanced Room-Temperature NO_2 Sensing Properties. *J .Alloys Compd.* **934**, 167950 (2023).

(24) Yuvaraja, S.; Surya, S. G.; Vijjapu, M. T.; Chernikova, V.; Shekhah, O.; Eddaoudi, M.; Salama, K. N. Fully Integrated Organic Field-Effect Transistor Platform to Detect and to Quantify NO2 Gas. *pss (RRL) – Rapid Research Letters* **14** (6), 2070027 (2020).

(25) Li, H.-Z.; Pan, Y.; Li, Q.; Lin, Q.; Lin, D.; Wang, F.; Xu, G.; Zhang, J. Rationally Designed Titanium-Based Metal-Organic Frameworks for Visible-Light Activated Chemiresistive Sensing. *J. Mater. Chem. A* **11**, 965 (2023).

(26) Kim, J. O.; Koo, W. T.; Kim, H.; Park, C.; Lee, T.; Hutomo, C. A.; Choi, S. Q.; Kim, D. S.; Kim, I. D.; Park, S. Large-Area Synthesis of Nanoscopic Catalyst-Decorated Conductive MOF Film Using Microfluidic-Based Solution Shearing. *Nat Commun.* **12,** 4294 (2021).

(27) Arul, C.; Moulaee, K.; Donato, N.; Iannazzo, D.; Lavanya, N.; Neri, G.; Sekar, C. Temperature Modulated Cu-MOF Based Gas Sensor with Dual Selectivity to Acetone and NO_2 at Low Operating Temperatures. *Sens. Actuators B Chem.* **329**, 129053 (2021).

(28) Mathankumar, G.; Harish, S.; Mohan, M. K.; Bharathi, P.; Kannan, S. K.; Archana, J.; Navaneethan, M. Enhanced Selectivity and Ultra-Fast Detection of NO_2 Gas Sensor via Ag Modified WO_3 Nanostructures for Gas Sensing Applications. *Sens. Actuators B Chem*. **381**, 133374 (2023).

(29) Small, L. J.; Henkelis, S. E.; Rademacher, D. X.; Schindelholz, M. E.; Krumhansl, J. L.; Vogel, D. J.; Nenoff, T. M. Near-Zero Power MOF-Based Sensors for NO_2 Detection. *Adv. Funct. Mater.* **30** (50) (2020).

(30) Koo, W. T.; Kim, S. J.; Jang, J. S.; Kim, D. H.; Kim, I. D. Catalytic Metal Nanoparticles Embedded in Conductive Metal–Organic Frameworks for Chemiresistors: Highly Active and Conductive Porous Materials. *Adv. Sci.* **6**,1900250 (2019).

(31) van Hoang, N.; Duc, L. M.; Hiep, N. T.; Hung, N. M.; Nguyen, C. v.; Hung, P. T.; Hoat, P. D.; Vo, V. K.; Heo, Y. W. Optimization of Synthesis Conditions and Sensing Performance of Electrospun $NiFe_2O_4$ Nanofibers for H_2S and NO_2 Detection. *J. Alloys Compd.* **936,** 168276 (2023).

(32) Ma, Y.-X.; Gao, B.; Li, Y.; Wei, W.; Zhao, Y.; Ma, J.-F. Macrocycle-Based Metal–Organic Frameworks with NO_2-Driven On/ Off Switch of Conductivity. *ACS Appl. Mater. Interfaces* **13**, 27066–27073 (2021).

(33) Zhan, M.; Hussain, S.; AlGarni, T. S.; Shah, S.; Liu, J.; Zhang, X.; Ahmad, A.; Javed, M. S.; Qiao, G.; Liu, G. Facet Controlled Polyhedral ZIF-8 MOF Nanostructures for Excellent NO2 Gas-Sensing Applications. *Mater. Res. Bull.* **136,** 111133 (2021).

(34) Pedrosa, J. M.; Dooling, C. M.; Richardson, T. H.; Hyde, R. K.; Hunter, C. A.; Martín, M. T.; Camacho, L. Characterization and Fast Optical Response to NO_2 of Porphyrin LB Films. *Mater. Sci. Eng. C* **22** (2), 433–438 (2002).

(35) Queirós, C.; Moscoso, F. G.; Almeida, J.; Silva, A. M. G.; Sousaraei, A.; Cabanillas-González, J.; Ribeiro Carrott, M.; Lopes-Costa, T.; Pedrosa, J. M.; Cunha-Silva, L. MOF-Based Materials with Sensing Potential: Pyrrolidine-Fused Chlorin at UiO-66(Hf) for Enhanced NO_2 Detection. *Chemosensors* **10**, 511 (2022).

(36) Gulino, A.; Mineo, P.; Scamporrino, E.; Vitalini, D.; Fragalà, I. Molecularly Engineered Silica Surfaces with an Assembled Porphyrin Monolayer as Optical NO2 Molecular Recognizers. *Chem. Mater.* **16** (10), 1838–1840 (2004).

(37) Gamonal, A.; Sun, C.; Mariano, A. L.; Fernandez-Bartolome, E.; Guerrero-Sanvicente, E.; Vlaisavljevich, B.; Castells-Gil, J.; Marti-Gastaldo, C.; Poloni, R.; Wannemacher, R.; Cabanillas-Gonzalez, J.; Sanchez Costa, J. Divergent Adsorption-Dependent Luminescence of Amino-Functionalized Lanthanide Metal−Organic Frameworks for Highly Sensitive NO_2 Sensors. *J. Phys. Chem. Lett.* **11**, 9, 3362-2268 (2020).

(38) Moscoso, F. G.; Almeida, J.; Sousaraei, A.; Lopes-Costa, T.; Silva, A. M. G.; Cabanillas-Gonzalez, J.; Cunha-Silva, L.; Pedrosa, J. M. Luminescent MOF Crystals Embedded in PMMA/ PDMS Transparent Films as Effective NO_2 Gas Sensors. *Mol. Syst. Des. Eng*. **5**, 1048 (2020).

(39) Schulz, M.; Gehl, A.; Schlenkrich, J.; Schulze, H. A.; Zimmermann, S.; Schaate, A. A Calixarene-Based Metal–Organic Framework for Highly Selective NO_2 Detection. *Angew. Chem. Int. Ed.* **57** (39), 12961–12965 (2018).

(40) Zhang, J.; Hu, E.; Liu, F.; Li, H.; Xia, T. Growth of Robust Metal-Organic Framework Films by Spontaneous Oxidation of a Metal Substrate for NO_2 Sensing. *Mater. Chem. Front.* **5**, 6476 (2021).

(41) Sava Gallis, D. F.; Vogel, D. J.; Vincent, G. A.; Rimsza, J. M.; Nenoff, T. M. NO_x Adsorption and Optical Detection in Rare Earth Metal−Organic Frameworks. *ACS Appl. Mater. Interfaces* **11**, 46, 43270-43277 (2019).
