# Supplementary material for: Synergistic binding sites in a metal-organic framework for the optical sensing of nitrogen dioxide
Source: Nat Commun. 2023 May 2;14:2506. doi: 10.1038/s41467-023-38170-9 (PMC10154382; doi:10.1038/s41467-023-38170-9)
Supplement: Supplementary file 3 — Description of Additional Supplementary Files [file 41467_2023_38170_MOESM3_ESM.pdf]

Description of Additional Supplementary Files

File Name: Supplementary Data 1
Description: Atomic coordinates of the optimized computational models.
